# Spatiotemporal patterns and drivers of orchard meadow loss in South Tyrol, Italy

**DOI:** 10.1038/s41598-024-81077-8

**Published:** 2024-12-28

**Authors:** Alexander Schönafinger, Lukas Egarter Vigl, Erich Tasser

**Affiliations:** 1 Institute for Alpine Environment, Eurac Research, Drususallee/Viale Druso 1, Bolzano/Bozen, 39100 Italy; 2 Department of Ecology, University of Innsbruck, Sternwartestrasse 15/Technikerstraße 25, Innsbruck, 6020 Austria

**Keywords:** Agroforestry, Cultural landscape, Land use/Land cover change, Temperate climate, Agroecology, Biodiversity, Ecosystem ecology

## Abstract

Orchard meadows, a specific agroforestry system characterised by scattered high-stem fruit trees, are a traditional element of several cultural landscapes in Central Europe and provide important ecosystem services. Since the middle of the 20th century, orchard meadows have drastically declined across Europe. Spatial information on the drivers and patterns of such a decline in several regions in Central Europe is lacking. In this study, we aimed to provide the first detailed insights into the development of orchard meadows in South Tyrol. We assessed the current distribution of orchard meadows in South Tyrol (Northern Italy) and analysed the patterns and drivers of their decline over the last 75 years. Aerial images of the orchard meadows in South Tyrol from the 1950s up to the 2020s were examined, and changes in their distribution were statistically analysed. The results revealed that the historical orchard meadow area in South Tyrol has decreased by more than 6,000 ha (−95%) over the past 75 years, showing one of the largest reductions in Central Europe. Orchard meadow loss was highest at lower elevations (–5,155 ha), shallow slopes (–4,313 ha), and expositions towards the south (–5,035 ha). Orchard meadows have declined due to agricultural intensification towards the establishment of modern fruit orchards (56%) and intensively managed grasslands (8%), urbanisation (23%), and reforestation (6%), and other types of land use/land cover (6.5%). Only 0.5% of the traditional orchard meadows have been preserved. The results suggest that orchard meadows are at a high risk of disappearing from the South Tyrolean landscape, resulting in the loss of genetic diversity of many local or indigenous fruit varieties (especially apples and pears). Immediate action is needed to safeguard the current orchard meadow population and appropriate management measures are needed to highlight the multiple values of orchard meadows and provide a valuable alternative to intensive arable farming.

**Supplementary Information:**

The online version contains supplementary material available at 10.1038/s41598-024-81077-8.

## Introduction

Agroforestry has been practiced in Europe for a long time. Agroforestry practices encompass the agricultural management of the understory in the presence of scattered, usually high-stemmed trees. These trees can be used either for forestry or for both forestry and agriculture^1^. Wood pasture, one of the earliest forms of agroforestry, can be traced back to the Neolithic Period^2^. Other ancient agroforestry systems such as ‘Dehesas’ in Spain and ‘Montados’ in Portugal, wherein pastures were integrated with oak woodlands, date back to 4,500 B.C.^3^. In Europe, agroforestry can be mainly categorized into silvoarable (arable or horticultural crops planted underneath trees) or silvopastoral (forage and livestock production in forests, woodland, or open forest trees) systems. A specialised form of silvoarable and -pastoral systems are orchard intercropping systems, where the understory management is associated with the cultivation of fruit trees^2,4^. Additionally, agroforestry systems can be encompassed by linear or small woody feature elements, such as riparian tree strips, avenue trees, or hedgerows^5^. Agroforestry systems are estimated to extend across a total area of over 10.6 million hectares in Europe, with approximately 1.2 million hectares dedicated to orchard intercropping systems^6^. Orchard meadows (also referred to as traditional orchards), also called ‘Streuobstwiese’ (German)^7^, ‘pré-verger’ (French), ‘luční sad’ (Czech), and ’sadová lúka’ (Slovak)^8^, is the prevalent type of orchard intercropping system implemented in European regions with temperate climate. Orchard meadows are characterised by high-stemmed (1.6–1.8 m) fruit trees such as apples, pears, plums, apricots, and cherries, and nut trees such as walnuts or chestnuts^7,9^ distributed across meadows, pastures, or arable land^10^. The tree density in orchard meadows typically ranges from 20 to 100 trees ha^− 1^^8,9^. The main principles of modern fruit cultivation practices, such as reproduction and grafting, were already applied in Ancient Greece and the Roman Empire; such practices were introduced to Central Europe by the Romans^7,11^. Orchards became a prominent element of the Central European landscape as early as the 17th century until the 18th and 19th centuries. During this period, government authorities played an important role in the expansion and rejuvenation of old orchard meadows by establishing nurseries and enacting policies such as granting citizenship and marriage license upon fulfilling the citizens’ obligation to plant fruit trees^7^. The economic motivation behind these policies promoted the development of traditional orchards^7,12^, which still were mostly silvoarable systems at this time^10,11^. It was only at the beginning of the 20th century when orchards were increasingly combined with grasslands in response to economic shift to dairy farming^7^.

Since then, traditional agroforestry systems, especially orchard meadows, have played key roles in providing habitats for various plant and animal species. These systems create ecological niches that support plants and animals from both forest and open-land communities as well as specialised species that rely on complex and varied structures within these ecosystems^13,14^. For example, orchard meadows provide habitat for a considerable number of specialised wild bees, such as solitary bees^15^. Furthermore, unlike intensively managed orchards, they are frequently associated with apiculture due to their limited exposure to pesticides^13,16,17^. In addition to their significant role in supporting biodiversity, orchard meadows deliver a multitude of ecosystem services (ES) such as preserving the genetic diversity of old fruit varieties, increasing the aesthetic value of landscapes, and providing habitats for pollinators^2,18^. A strong interconnection between ecological and sociocultural values also exists within traditional agroforestry systems^19^, making them substantial contributors to human well-being. However, the socioeconomic developments in the 20th century have led to a dramatic decline in orchard meadows and their associated ecosystem services and, thus, have negatively affected cultural heritage through the loss of numerous traditional fruit varieties^20^. Most of the modern fruit varieties and agricultural practices associated with fruit cultivation and propagation originated in the Persian, Egyptian, and Indian Empires. Over time, several fruit varieties have been developed. For example, there are 2,504 apple, 1,623 pear, and 1,696 plum/damson varieties in Switzerland; 1,067 apple, 168 pears, and 1,000 plum/damson varieties in Germany; and approximately 2,000 apple, 1,500 pear, and 1,000 plum/damson varieties in Austria^21^. Along with agricultural intensification and modernisation in the 20th century, fruit varieties cultivated in orchard meadows were standardised due to exchanges, trade, and variety recommendations across Central Europe^20^, leading to the declining importance of traditional orchard varieties in general and that of regional and local varieties in particular^10,22^. The decrease in the area occupied by orchard meadows ranges from 15% in Bohemia, Czech Republic^8^, to 94% in Belgium^7^. Other fruit-intercropping agroforestry systems in Europe, such as the cider orchards in Great Britain (−56%), have also suffered significant losses in terms of traditional orchard area^23^.

The historical evolution of fruit cultivation in the Autonomous Province of South Tyrol (Südtirol/Alto Adige, northern Italian province), which is reported in the literature, indicates a spatiotemporal development comparable to that observed in Central Europe^13^. The most prevalent agroforestry systems in this region are larch meadows^24,25^ and chestnut groves^26,27^; however, orchard meadows also form an integral part of the cultural landscape in South Tyrol^13,28^.

Nevertheless, the impact of these historical developments on the past and present spatial distribution of orchard meadows in South Tyrol remains largely unknown. This knowledge gap can be partly attributed to agricultural statistical assessments that predominantly concentrate on intensive agricultural systems. However, numerous studies have omitted the inclusion of South Tyrol in the broader context of Central Europe^2,8,10^. In this study, we aimed to provide the first detailed insights into the development of orchard meadows in South Tyrol. In view of the development of agriculture in Central Europe, we hypothesised that South Tyrol has experienced a significant reduction in orchard meadow areas since the middle of the 20th century. To test this hypothesis, we quantitatively assessed the spatiotemporal dynamics of changes in orchard meadow areas since the mid-20th century and identified the key drivers behind their transformations.

## Results

### Spatiotemporal development of orchard meadows

**Fig. 1:**
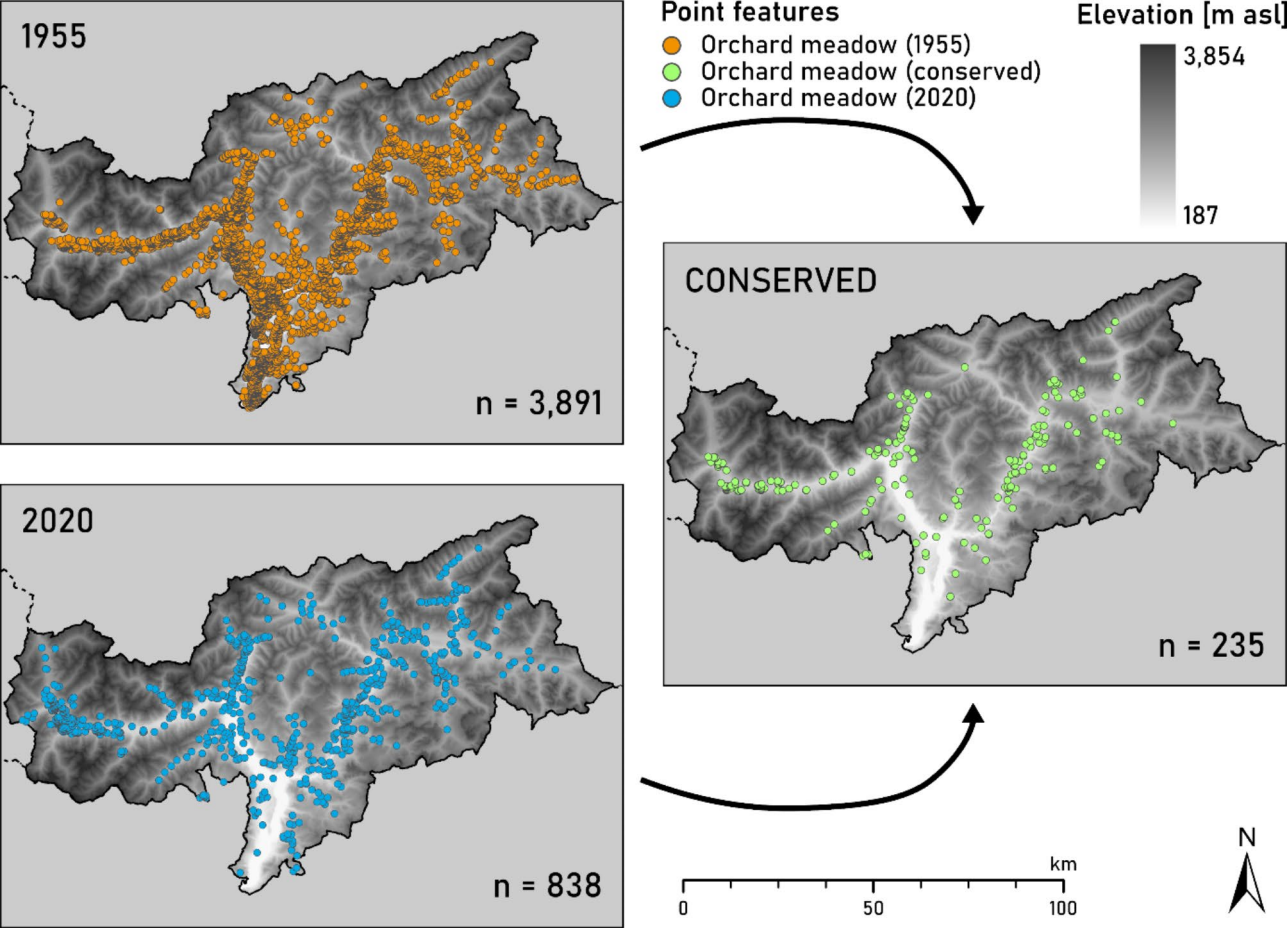
Distribution of orchard meadows in South Tyrol in 1955 (6,365 ha) and 2020 (296 ha) as well as the distribution of conserved orchard meadows (33 ha). The conserved map segment shows the orchard meadows present in both 1955 and 2020. The points represent the centre of corresponding orchard meadow polygons. The orchard meadow count (n) is displayed in the bottom-right corner of each map segment. The elevation gradient layer has been generated using the Digital Elevation Model of South Tyrol dataset^29^. The compilation of maps was generated using ArcGis Pro 3.3.1^30^.

The number of contiguous orchard meadows in South Tyrol has decreased by approximately 3000 single fields (−78%) (Fig. 1) over the last 75 years. Although this decline was consistent across all districts, the highest losses (96% reduction) were observed in Überetsch-Südtiroler Unterland and Bozen; in contrast, the smallest reduction (48%) was observed in Vinschgau.

**Fig. 2:**
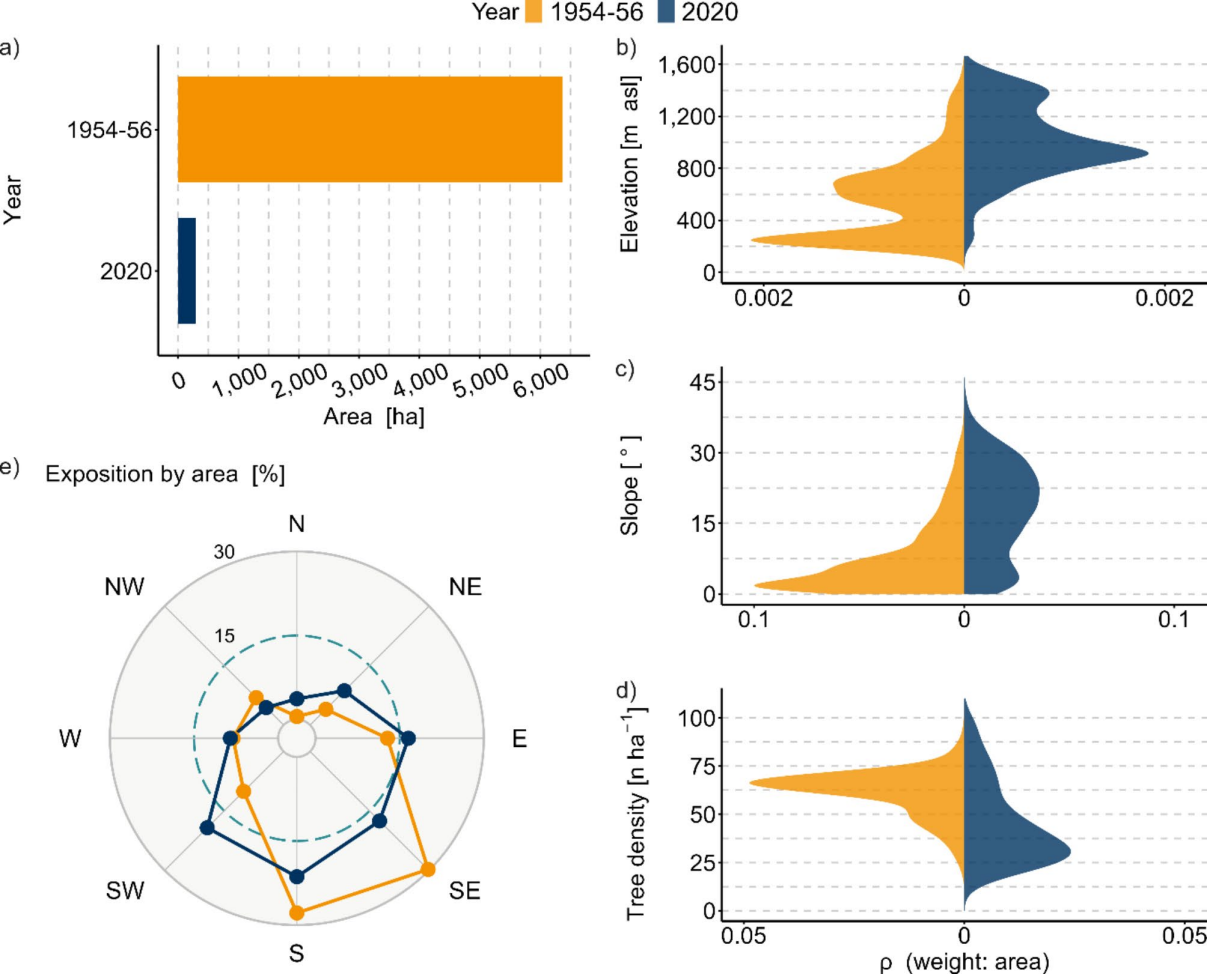
Changes in the total area of orchard meadows from the 1950s (**a**) and relative distribution by elevation (**b**), slope (**c**), tree density (only for the district of Vinschgau) (**d**), and exposition (**e**).

In the 1950s, the total area covered by orchard meadows was 6,365 ha, contributing to 6.6% of agricultural land in favourable areas (UAA) in 1954. Over the past 75 years, it has decreased by over 95% (−6,069 ha), leaving only 296 ha (0.3% of the UAA in 2010) today (Table 1; Fig. 2a; Supplementary Table S4). Bozen (−99.5%) and Überetsch-Südtiroler Unterland (−99.4%) suffered the most significant losses, whereas Wipptal (−82.7%) and Pustertal (−79.2%) suffered the least.

**Table 1:** Historical and recent distribution of orchard meadows within South Tyrol (bold) and its districts.

	Area (ha)		Area change (%)
	1955	2020	
Bozen	320.2	1.6	−99.5
Burggrafenamt	1,964.1	65.5	−96.7
Eisacktal	497.4	41.8	−91.6
Pustertal	250.2	52.1	−79.2
Salten-Schlern	211.3	35.6	−83.1
Überetsch-Südtiroler Unterland	1,637.5	10.2	−99.4
Vinschgau	1,455.8	84.7	−94.2
Wipptal	28.5	5.0	−82.7
**South Tyrol**	**6,364.9**	**296.4**	**−95.3**

The largest contiguous orchard meadow in the 1950s covered approximately 340 ha; however, the area of the largest traditional orchard in 2020 shrank to only 3 ha (Supplementary Fig. S1). At the same time, the average area of orchard meadows has decreased from 0.54 ± 0.38 to 0.25 ± 0.14 ha (Welch’s t-test: t_4003_ = 39.7, *p* < 0.001) (Supplementary Fig. S3a). This trend was consistent across all districts, especially for Bozen, Überetsch-Südtiroler Unterland, and Burggrafenamt, which showed the most substantial changes (Supplementary Fig. S4). Finally, the mapping inaccuracy was less than 2% of the total area following cross-referencing a comprehensive agricultural land use map with the 2020 orchard meadow dataset.

The tree density observed in the district of Vinschgau was frequently higher in historic orchard meadows (Fig. 2d). On average, it declined from 56 ± 19 trees ha^− 1^ to 45 ± 23 trees ha^− 1^ (Welch’s t-test: t_374_ = 3.5, *p* < 0.001) (Supplementary Fig. S3b). In current orchard meadows, only the districts of Bozen and Überetsch-Südtiroler Unterland, which together contribute to just 4% of the current orchard meadow area (Table 1), show a higher average tree density than the historic mean in Vinschgau (Supplementary Fig. S5).

Moreover, there was a reduction in the total area of orchard meadows across all elevation belts. During the 1950s, approximately 82% of the orchard meadow area was situated at elevations below 800 m asl (colline belt). Currently, however, orchard meadows are predominantly located in the submontane belt (800–1,200 m asl), constituting 52% of the total area (Fig. 2b; Supplementary Table S3a). Thus, the most significant reduction in orchard meadow area occurred in the colline belt (−98.8%), followed by those in the submontane belt (−82.3%) and the montane belt (−71.2%) (Supplementary Table S3a, Fig. S8). Notably, the average elevation in Salten-Schlern has remained relatively stable, indicating a consistent decrease across all elevations. Conversely, the average elevation in Wipptal decreased, indicating a more pronounced reduction in orchard meadow area at higher elevations (Supplementary Fig. S6).

Regarding the distribution of orchard meadows based on slope, 76% of the orchard meadows in the 1950s were situated on surfaces with gentle inclinations (≤ 11°). In contrast, 74% of the orchard meadows are currently distributed along moderate and steep slopes (> 11°) (Fig. 2c; Supplementary Table S3b, Fig. S9). The decline in the distribution of orchard meadows gradually diminished as the slope increased; thus, the areas with the steepest slopes had the lowest reduction in the distribution of orchard meadows. This trend was consistent across all districts; Überetsch-Südtiroler Unterland, Burggrafenamt, and Bozen exhibited the most significant changes, whereas Salten-Schlern showed the smallest changes (Supplementary Fig. S7).

The distribution of orchard meadows by exposition decreased toward the southeast and south; thus, more orchard meadows are now facing southwest (Fig. 2e; Supplementary Table S3c, Fig. S10).

### Land-use/land-cover change

**Fig. 3:**
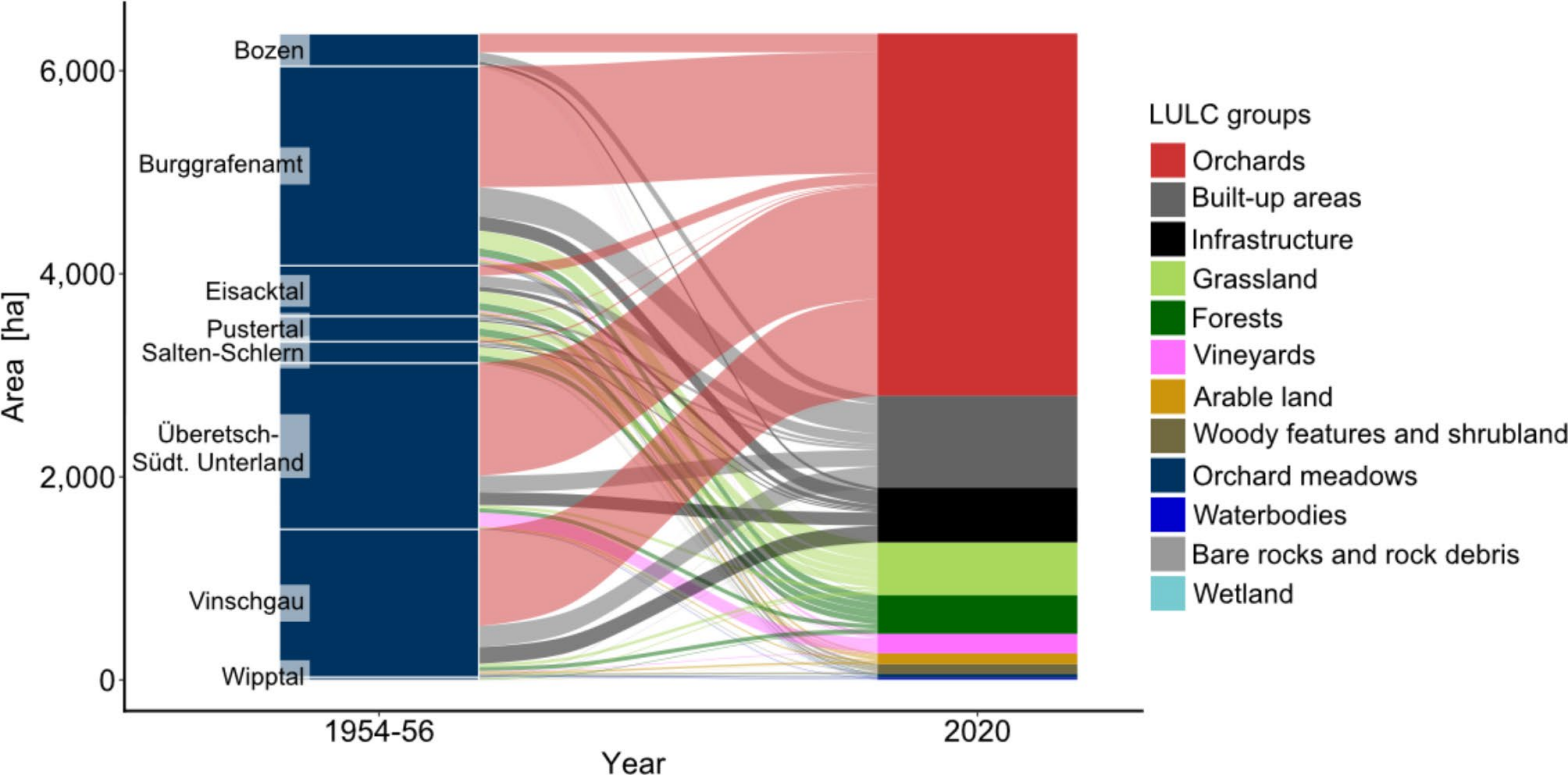
Transformation of historical orchard meadows into current land-use/land-cover (LULC) in the districts of South Tyrol.

The loss of historical orchard meadows in South Tyrol was due to their conversion into modern orchards (56%), built-up areas (14.3%), infrastructure (8.4%), grasslands (8.2%), and forests (6%) (Fig. 3; Supplementary Table S6, Fig. S12). Only a small portion of the orchard meadows (0.5%) (Fig. 1; Supplementary Table S6) has been retained since the 1950s. In summary, 68.8% of the area was transformed into intensive agricultural land-use/land-cover (LULC) types (arable land, intensively used grasslands, orchards, and vineyards). Urbanisation (22.7% occupied by built-up areas and infrastructure) and land abandonment (7.7% occupied by forests, woody features, and shrublands) also contributed to significant LULC changes.

There were regional variations in LULC changes among the districts of South Tyrol. The orchard meadows in Überetsch-Südtiroler Unterland (68%), Vinschgau (66%), Bruggrafenamt (61%), and Bozen (57%) were mostly converted into modern orchards. Meanwhile, the orchard meadows in Eisacktal were mainly transformed into grasslands and built-up areas, whereas those in Pustertal, Salten-Schlern, and Wipptal were converted into grasslands and forests (Fig. 3; Supplementary Table S6).

## Discussion

### Changing cultural landscape

Orchard meadows are the most recent agroforestry system implemented in South Tyrol; they emerged in response to the Napoleonic War to enhance food security after the 1820s^11^. Orchard meadows covered approximately 6,400 ha in 1955; however, the area covered by orchard meadows in the following years subsequently declined by 95%, which is significantly above the Central European average of 75%^31^ (Table 2). The decline in the total area of orchard meadows in Central Europe is mainly driven by agricultural intensification, urbanisation, and land abandonment^8–10,12,32^. Our study revealed a similar trend in South Tyrol, where orchard meadow loss is primarily attributed to agricultural intensification, leading to the transformation of over half of the area into modern orchards with densely planted, small-sized trees. The separation of fruit and fodder production has been strongly promoted in South Tyrol since the 1920s through the use of pesticides in fruit production as they have been adversely affecting the health of livestock^11^. Other contributing factors include increased mechanisation^19,33^, fertiliser usage^2,7^, and production costs^24,32^, changes in farming practices, transition from full-time to part-time farming, and reduction in household size and available family labour resources^10,24^.

**Table 2:** Literature collection of the decline in orchard meadows in eight European countries and in cider orchards (silvoarable system similar to orchard meadows) in Britain. All listed countries are situated in the temperate climate zone^10^. * marks countries that entirely belong to Central Europe and ** mark countries that are partially belonging to Central Europe.

Country / Territory		Time period	Change (–%)	Reference
Belgium**		1944–1976	94	^7^
Luxembourg*		1902–1993	78	^7^
Germany*		Since 1950s	75	^34^
North-west		1979–2009	74	^8^
South		1965–2005	48	^9^
East		1964–2008	46	^9^
South-west		1968–2009	22	^8^
Hessen		1938–1983	92	^7^
North Rhine-Westphalia		1951–1990	92	^7^
Hamburg		1951–1965	87	^7^
Rhineland-Palatinate		1951–1990	84	^7^
Niedersachsen		1951–1965	76	^7^
Bremen		1951–1965	71	^7^
Baden-Württemberg		1938–1990	70	^35^
Thuringia		1981–1988	67	^7^
BRD-West		1951–1982	65	^7^
Saarland		1965–1988	61	^7^
Bavaria		1951–1965	33	^7^
Switzerland*		1954–1991	70	^10^
Austria*		1960–1984	65	^7^
Burgenland		19th century	85	^36^
Mostviertel		1953–2002	70	^32^
Britain		1950–2007	56	^23^
England		1950–2007	81	^23^
Wales		1950–2007	81	^23^
France**		1982–2003	44	^9^
Slovakia*				
Central		Since 1950s	75	^8^
Czech Republic*				
Bohemia		Since mid-1950s	15	^8^

Moreover, the transformation of historical orchard meadows in South Tyrol was notably influenced by urbanisation. Since the 1950s, the population has surged by a factor of 3.2 (from 168,301 in 1951 to 532,616 inhabitants in 2020)^37^. Consequently, the expansion of built-up areas has become necessary, leading to the removal of several orchard meadows that are often situated in belts around settlements^9^. Urbanisation also involves the expansion of infrastructure networks, including roads and railways^38^. A key development was the construction of the A22 highway in 1972, which, coupled with the adoption of new plantation and conservation technologies, further facilitated specialisation in intensive fruit production, particularly after the 1970s^11,28^. Such technologies involved, for example, an even more compact plantation of trees with up to 10,000 trees ha^− 1^ or more advanced cooling techniques for fruit storage^11,39^.

A small portion of the orchard meadows in South Tyrol has been abandoned and reforested. Land abandonment typically occurs in areas with less favourable topographic conditions, characterised by high elevations, steep inclinations, and north-facing slopes^40,41^, whereas agricultural intensification and urbanisation mainly occur in areas with more suitable topographic conditions (low elevation and inclination and south-facing slopes)^38^.

The results of this study, along with those of other studies, demonstrate that the development of the landscape in South Tyrol has resulted in the displacement of traditional cultural elements, such as orchard meadows, into less favourable agricultural areas. Such areas are frequently inaccessible for agricultural machinery, leading to a general association with an extensive type of cultivation^24,25^. In contrast, areas that are cultivated extensively in topographically favourable locations frequently find themselves in competition with intensive agriculture, as these areas are more accessible and suitable for the use of machinery, allowing for more intensive farming practices. Furthermore, the suitable climatic conditions facilitate intensive farming, particularly in the valley floors at elevations up to approximately 1,000 m asl^38^. However, the rising temperatures associated with climate change may allow intensive agriculture to advance further into the montane vegetation belt, potentially displacing traditional and extensive agriculture into even less favourable areas or even eliminating some traditional agricultural practices entirely.

### Ecological and social consequences

Agroforestry systems, such as orchard meadows, offer numerous ecosystem services (ES) including provisioning, regulation and maintenance, and cultural services (Supplementary Table S8). Accordingly, every change in the land-use/land-cover (LULC) results in alterations in ES provisions^42^. The loss of orchard meadows due to the intensification of agriculture, urbanisation, and land abandonment primarily leads to a reduction in landscape heterogeneity and loss of the cultural, historical, and aesthetic values of a landscape element^7,38,41,43^. Moreover, agroforestry systems are generally considered beneficial for climate change mitigation and adaptation in agriculture, as the presence of perennial woody plants on agricultural land increases aboveground and belowground carbon stocks and simultaneously leads to a reduction in temperature and improves the water supply of the crops below^44,45^. Furthermore, orchard meadows are recognised as biodiversity hotspots^7,8,46^. For example, extensively managed orchard meadows in South Tyrol exhibit significantly higher taxonomic richness and diversity of soil macro-invertebrate communities as well as greater diversity of vascular plants, grasshoppers, butterflies, and birds than intensively managed orchards^13,15,47^.

Guariento et al. (2024) reported that orchard meadows in South Tyrol harboured endangered and/or rare species^15^ such as *Allium vineale* and *Orobanche lutea* (vascular plants), *Pachytrachis striolatus* and *Meconema thalassinum* (grasshoppers), *Melitaea didyma* and *Lycaena tityrus* (butterflies), and *Lanius collurio* and *Emberiza cia* (birds). The study further identified 77 bee species in orchard meadows, including a significant proportion of solitary and specialised bees. In contrast, only 29 bee species were found in intensive apple orchards^15^. As orchard meadows need pollinators and support them, they are often paired with beekeeping^10,16,48^. However, the high abundance of honeybees (*Apis melifera*) in orchard meadows may lead to the displacement of wild bee species^16^. Thus, orchard meadows offer a viable restoration solution in agriculture to support the recovery of degraded, damaged, or destroyed ecosystems and reintroduce more nature and biodiversity to the cultural landscape^46^. Furthermore, they could play a central role in the implementation of the EU Nature Restoration Law (Regulation 2022/869) and the EU Biodiversity Strategy for 2030 (COM/2020/380).

Another essential aspect of ES in orchard meadows is the genetic diversity of native fruit species, which contributes to cultural ecosystem services. Apple and pear trees are commonly found in traditional orchards. At the beginning of the 20th century, South Tyrol was still commercially producing approximately 200 apple and pear varieties^28^. Furthermore, 0.17 million tonnes (Mt) of apples and 36,860 t of pears were harvested in 1955^49^. However, the current fruit production landscape has changed dramatically in favour of intensive apple production^49^, yielding an annual harvest of approximately 1 Mt (peak of 1.2 Mt in 2014) and focusing primarily on just 11 apple varieties (77% of total production in 2022), with ‘Golden Delicious’ being the most predominant (25% of the total apple cultivation area)^50^. Moreover, pear production has significantly diminished, contributing to only 432 tons of the annual harvest of pomaceous fruits (predominantly ‘Williams Christ’, 70% of the pear harvest)^50^. In summary, pomaceous fruit production has increased by approximately 500%, apple production by 600% and pear production has decreased by 98.8% since the 1950s. As a consequence, the intensification of fruit production has led to the depletion of genetic diversity in favour of the mass production of apples; hence, orchard meadows constitute a refugium for autochthonous fruit varieties. ‘Initiative Baumgart’, for example, collected a list of fruit varieties that can be found in orchard meadows in South Tyrol^51,52^. This list includes over 80 apple, 40 pear, and 10 apricot, plum, and damson varieties (Supplementary Table S9).

### Management considerations for the preservation of orchard meadows

Nature conservation and intensive agriculture are antagonistic to each other^53^. Over the last 50 years, European agricultural policies regarding orchard conservation^54^ have pursued different concepts and objectives as a result of socioeconomic changes^55^. Agricultural goals and subsidies have often prevailed over nature conservation, resulting in endangered orchards and other species-rich and high-quality semi-natural habitats^55^. Therefore, the coexistence of agricultural production and nature conservation should be improved through comprehensive political coordination, including harmonised spatial planning. Environmental subsidies and farmers’ moral judgements are probably the most important factors^56^ that would facilitate a paradigm shift supporting both agricultural sector and natural conservation. Nevertheless, it can be observed that the majority of farmers in Central Europe are primarily focused on maximising their income. It is only in circumstances where support has a significant impact on this goal that it can have a tangible influence on the decisions of farmers^57^. Under the ‘programme for landscape conservation’ in South Tyrol, subsidies worth a premium of 550€ ha^− 1^ are exclusive for agriculturally managed orchard meadows and chestnut groves^58^. These subsidies entail additional administrative requirements, including a minimum tree canopy of 20%, a minimum area of 0.36 ha, and a commitment to maintaining the orchards for a minimum of 5 years. Furthermore, the use of mineral or liquid fertilisers and pesticides is prohibited, and the removal of fallen branches and competing shrubs is mandatory. Of the approximately 500 sites that have been enrolled in this subsidy program in 2021, the majority were chestnut groves and only 20 were orchard meadows^59^. The existing programme may lack the capacity to sufficiently address the financial requirements for cultivating orchard meadows while simultaneously meeting the necessary conditions for landscape conservation. This becomes particularly apparent considering that 99% of the mapped traditional orchards did not submit applications for subsidies. In contrast, Switzerland has subsidy programs for high-stemmed fruit trees in agroforestry systems, wherein farmers receive annual support ranging from 13.5 to 31.5 CHF per tree^60^ (e.g., 80 trees ha^− 1^: 1,080 to 2,520 CHF ha^− 1^ a^− 1^). Additionally, various organisations provide financial support to actively promote the establishment of agroforestry systems^61^. For instance, the association ‘Hochstamm Suisse’, in collaboration with ‘Stiftung myclimate’, currently supports the replanting and maintenance of high-stem trees with 105 CHF per tree^62^. Indeed, financial support, coupled with favourable market prices, enhances the economic competitiveness of agroforestry systems, including orchard meadows^63^. Accordingly, 30% of the former orchard meadows still exist today^10^.

For most orchard meadow owners, factors other than subsidies affect their agricultural practices. Despite these challenges, some farmers in South Tyrol sell harvested fruits as sustainable regional products to local fruit associations, gastronomic facilities, or farmers’ markets. Moreover, the orchard meadows in South Tyrol are integrated with agrotourism to provide visitors with aesthetic and recreational landscape elements; occasionally, the orchard meadows serve as a retreat for guests participating in the ‘holiday on a farm’ program^64^. In the context of sustainable land use, the quantity of ES provided by orchard meadows is more significant than their economic efficiency (Herzog 1998). Local initiatives such as Sortengarten Südtirol^65^ and Initiative Baumgart^51^, are actively working to raise awareness among the public, local stakeholders, and administrative authorities about the importance of the functionalities and ES offered by orchard meadows.

Despite such initiatives, orchard meadows face economic challenges compared with modern agricultural systems in South Tyrol. Consequently, they remain under constant pressure and are at a high risk of extinction because of the ongoing intensification of agriculture and unfavourable economic conditions^7,11,12,28^.

### Methodological limitations

The mapping process employed in this study is subject to inaccuracies. These issues include discrepancies in the digital orthophotos utilised, which exhibit variations in resolution and quality, including pixel size and colour spectrum. The orthophotos did not perfectly align in all areas, leading to small discrepancies, primarily attributed to georectification issues. In addition, both orthophotos were influenced by factors such as topography, camera angle, and illumination conditions.

In the historical orthophoto, identifying orchard meadows proved challenging in darker areas, and distinguishing fruit trees from other tree cover was more difficult because of grayscale tones. Conversely, the current orthophotos present challenges in identifying potential orchard meadow areas shaded by nearby forests or buildings, which may have resulted in some areas being overlooked. Nevertheless, identifying fruit trees during the current time step was easier and occasionally aided by detailed tree shadows under suitable light conditions. There may have been instances of confusion between fruit trees and other deciduous tree cover, potentially leading to an error rate similar to that observed in the historical distribution (personal estimation: < 1% average error rate).

Although on-site verification of the identified orchard meadows could enhance mapping accuracy, it was impractical in this study because of resource limitations. In addition, verification is only feasible for the current distribution, introducing potential challenges in maintaining comparability between datasets. Nonetheless, periodic monitoring of the orchard meadow distribution is essential to safeguard the persistence of this rare LULC type. The verification method employed for the existing orchard meadows involved cross-referencing a detailed agricultural land use map, resulting in a mapping inaccuracy of less than 2%. However, no polygons were excluded from the 2020 dataset in order to maintain comparability between the two analysed datasets, due to the unavailability of a detailed agricultural land use map for the years 1954 to 1956.

## Materials and methods

### Study area

**Fig. 4:**
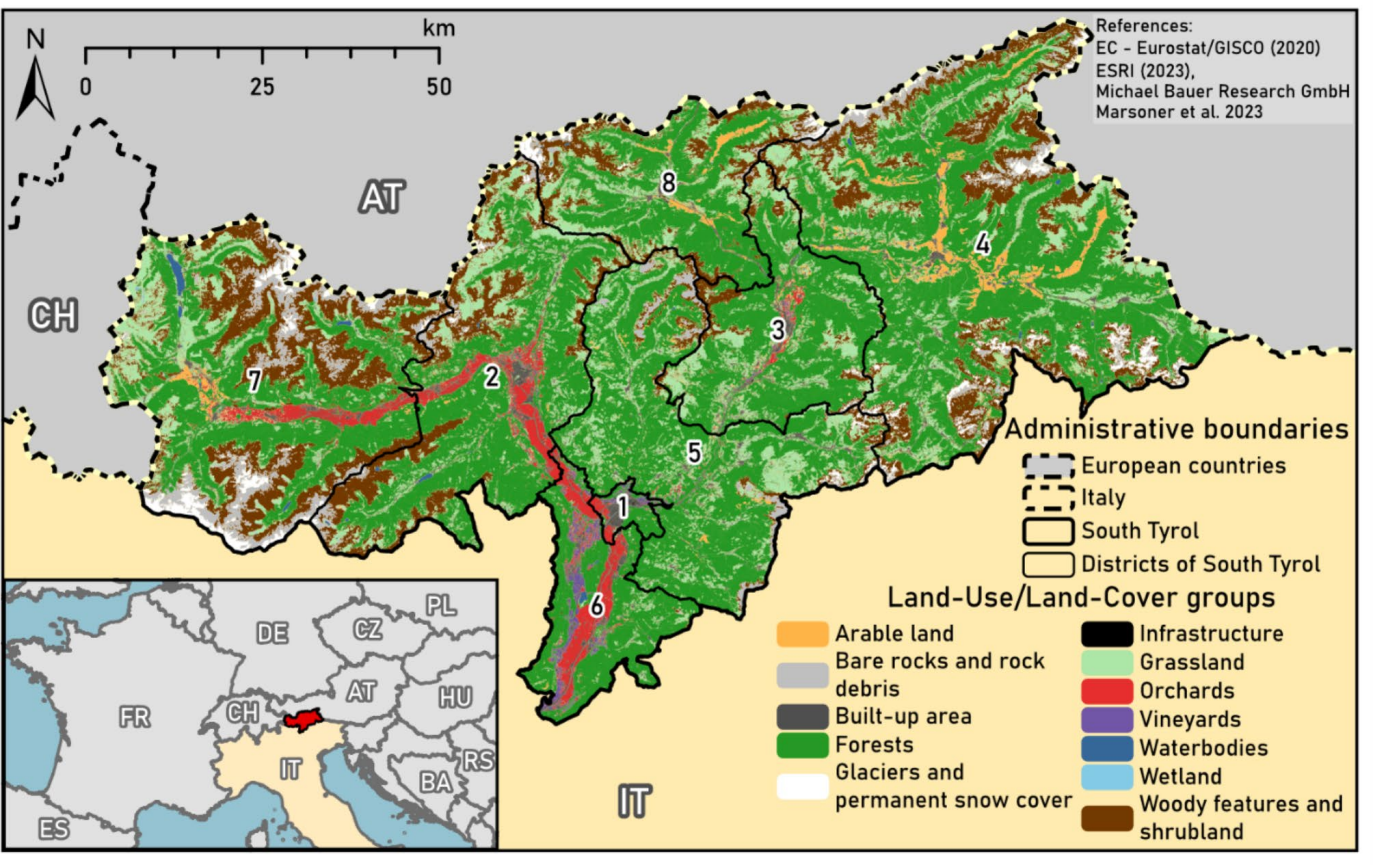
South Tyrol, the study area, is the northernmost province of Italy (IT) and is adjacent to Switzerland (CH) and Austria (AT). It is divided into eight districts: Bozen (1), Burggrafenamt (2), Eisacktal (3), Pustertal (4), Salten-Schlern (5), Überetsch-Südtiroler Unterland (6), Vinschgau (7), and Wipptal (8). The map was generated using ArcGis Pro 3.3.1^30^.

This study focused on the orchard meadows in South Tyrol (Südtirol/Alto Adige; 10.37–12.51°E and 47.12–46.19°N), the northernmost province of Italy. South Tyrol covers a total area of 7,400 km² and is organised into eight districts (Fig. 4). It is characterised by a continental climate with western Atlantic and Mediterranean influences; however, the climate in South Tyrol varies greatly due to its location in the Southern and Central Alps and large differences in its elevation, ranging from 180 to 3,900 m above sea level (asl)^66^.

The cultural landscape in South Tyrol has drastically changed since the middle of the 19th century, when it still belonged to the County of Tyrol. During this period, most of the broad valley floors were occupied by arable land in response to the Napoleonic War and subsequent famine and grain shortage^11,54^. The crops were then increasingly interspersed with fruit trees to improve the self-sufficiency of South Tyrol residents. The construction of railways, first linking Bozen (Italy) and Innsbruck (Austria) in 1867 and then extending between Bozen and Mals (Italy) in the early 20th century, opened new opportunities for the export of South Tyrol’s goods^67^. In the second half of the 19th century, fruit cultivation became increasingly important for earning additional income. In the 1920s, fruit cultivation began gaining prominence in the South Tyrolean region (which became an Italian province following World War I) and grassland, arable land, and vineyard areas were converted into traditional orchards^68^. Concurrently, the tree density in orchard meadows gradually increased, leading to the subsequent separation of fruit, crop, and grassland production. This shift was further accelerated by concerns over the adverse effects of pesticides on cattle^11^. Finally, the construction of Highway A22 in 1972 and the subsequent high demand for fruit exports (mainly apples) intensified fruit production in South Tyrol^11,39^.

Currently, the landscape of South Tyrol is characterised by forests (50%), agricultural land (37%), marginal land (10%), and settlement areas (3%). The agricultural land is further divided into pastures (59%), hay meadows (27%), orchards (8%), vineyards (3%), and arable land (3%)^69^. Notably, the agricultural land in Bozen and Überetsch-Südtiroler Unterland, is predominantly occupied by apple plantations and vineyards, whereas permanent hay meadows and pastures are the predominant land-use types in the other districts^70^. The total area of agricultural land used in favourable sites (Supplementary Table S1) has only slightly changed since the middle of the 20th century; however, there has been large-scale intensification of land use.

### Mapping approach of orchard meadows

**Fig. 5:**
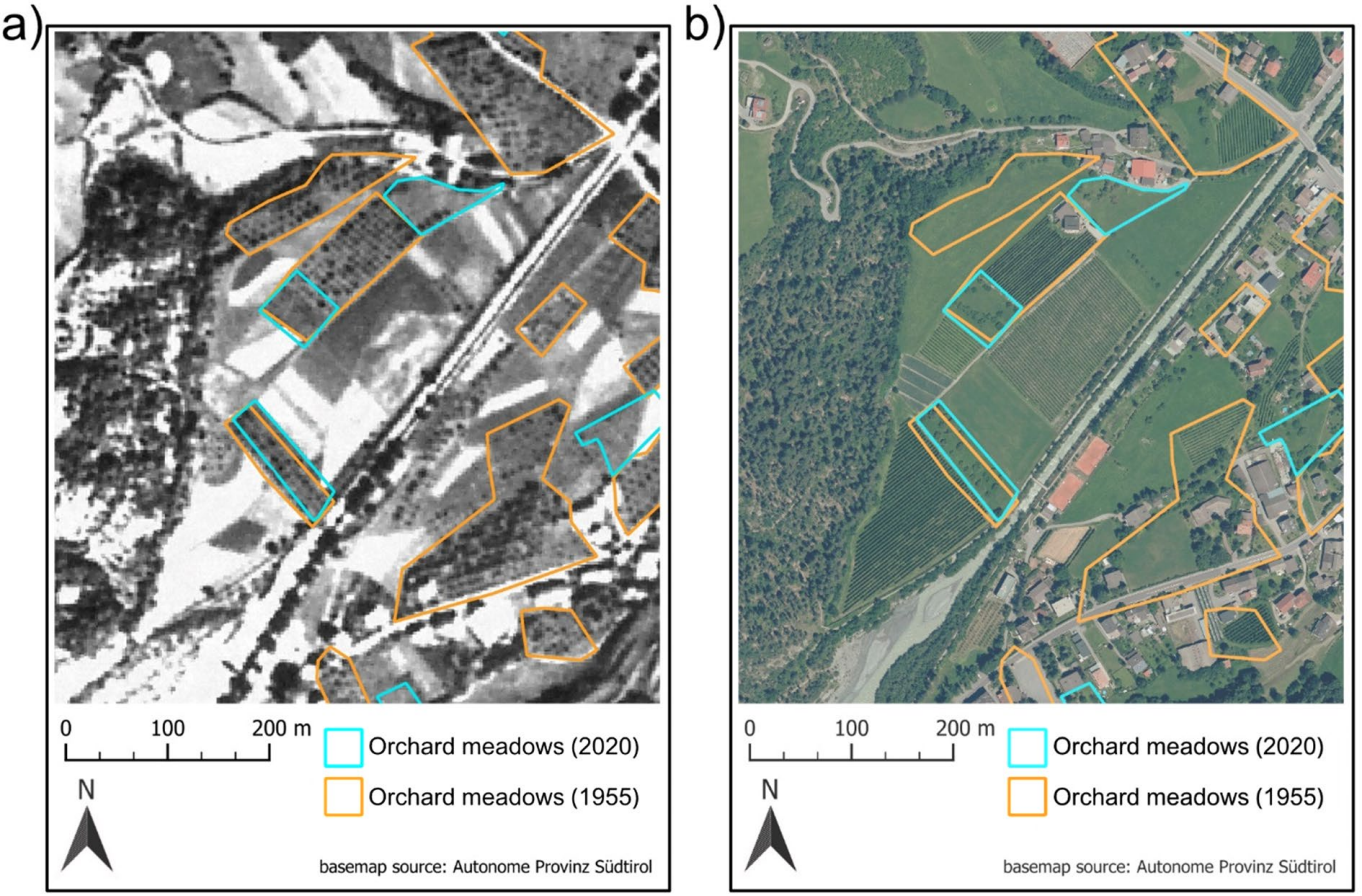
Example of the mapping approach using orthophotos of 1954–56^29^ (resolution: 1.5 × 1.5 m) (**a**) and 2020^29^ (resolution: 0.2 × 0.2 m) (**b**) in detail at Prad am Stilfserjoch (Vinschgau; 10.584635°E, 46.616560°N). The compilation of maps was generated using ArcGis Pro 3.3.1^30^.

Orchard meadows are defined in this study as grasslands containing tall fruit trees arranged either regularly or irregularly, with a minimum distance of 5 m between the edges of their canopies^40,52^. Moreover, orchard meadows should have a minimum area of 0.1 ha and a tree density of 20–100 trees ha^− 1^^9^. For spatiotemporal analysis, the historical and current distribution of orchard meadows in South Tyrol was mapped, following an approach similar to that of Plieninger et al. (2015)^9^. Single orchard meadows were manually mapped at a working scale of 1:2,500 using orthophotos captured in 1954–1956 (resolution: 1.5 × 1.5 m) and in 2020 (resolution: 0.2 × 0.2 m)^29^. Polygon boundaries were delineated based on visible field margins or around the outermost trees (Fig. 5). In order to validate the mapping process of currently existing orchard meadows, the recorded polygons were cross-referenced with the South Tyrol land use cadastre for agriculture and forestry of the Autonomous Province of South Tyrol (LAFIS) from 2020^59^. Furthermore, trees within the orchard meadows were recorded as points at each time step to assess tree density. Given the extensive number of orchard meadows in the 1950s, the historical point dataset exclusively covered trees within the district of Vinschgau as it showed the most resilient development of orchard meadows. Tree densities for recent time steps were mapped in all orchard meadows.

### Drivers of changes

The spatial and topographic distribution of orchard meadows was temporally compared to gain a deeper understanding of their development in South Tyrol. The elevation, slope, and exposition of South Tyrol, which were determined using the digital elevation model of South Tyrol, were analysed as potential site parameters^66^.

To determine the trend towards which orchard meadows have developed since the 1950s, land-use/land-cover (LULC) changes within the historical areas were analysed. The current LULC dataset^71^ was updated based on the current distribution of mapped orchard meadows. The resulting LULC dataset featured a pixel size of 5 m × 5 m and encompassed 52 different LULC categories in South Tyrol, which were aggregated into 12 main groups (Supplementary Table S5). Changes were assessed for South Tyrol as a whole and its individual districts.

### Data handling

The map datasets were calculated and modified using ArcMap v10.7^72^. The raw data were evaluated and the results were visualized in RStudio v2023.12.1.402^73^ with R v4.3.1^74^ (Supplementary Table S7). Figures containing maps were generated using ArcGis Pro v3.3.1^30^.

Spatial and temporal differences were subjected to statistical testing. Levene’s test^75^ was performed to assess homoscedasticity (package ‘psych v2.4.1’)^76^. The means of two independent samples were compared by employing Welch’s t-test^77^ for heterogeneous variances and Student’s t-test^78^ for homogeneous variances.

## Conclusion

Orchard meadows face severe risks of disappearing entirely from the cultural landscape of South Tyrol. With a decline of 95%, they marked one of the most substantial recorded declines in Central Europe since the middle of the 20th century. Despite serving as genetic reservoirs for numerous fruit varieties, particularly apples and pears, their potential decline is exacerbated by agricultural intensification. Orchard meadows play a crucial role in supporting biodiversity and providing ecosystem services, thus offering an opportunity to enhance the ecological sustainability of the cultural landscape in South Tyrol. Recognising the importance of extensive management within the implementation and realisation of the EU Nature Restoration Law and the EU Biodiversity Strategy for 2030 is important for orchard meadow conservation and the provision of various ecosystem services, especially habitat and biodiversity support, as well as climate mitigation. However, current regional subsidy programs focus solely on conserving valuable landscape elements, and do not address the economic competitiveness of traditional agroforestry systems. Regarding the subsidy standards in Switzerland and given the average tree density, a typical South Tyrolean orchard meadow should be annually supported by at least 620 to 1,450€ ha^− 1^ in order to reach economic sustainability. Consequently, there is a pressing need to give orchard meadows social value, intensify monitoring efforts, and incorporate orchard meadows into upcoming agricultural censuses to facilitate conservation strategies, identify key challenges in promoting their expansion, and identify the spectrum of fruit species to preserve the genetic diversity of autochthonous varieties.

## Electronic supplementary material

Below is the link to the electronic supplementary material.

Supplementary Material 1

## Data Availability

The datasets generated and/or analysed during the current study are available from the corresponding author on reasonable request.
