# Supplementary material for: Spatiotemporal patterns and drivers of orchard meadow loss in South Tyrol, Italy
Source: Sci Rep. 2024 Dec 28;14:30812. doi: 10.1038/s41598-024-81077-8 (PMC11680714; doi:10.1038/s41598-024-81077-8)
Supplement: Supplementary file 1 — Supplementary Material 1 [file 41598_2024_81077_MOESM1_ESM.docx]

**Spatiotemporal patterns and drivers of orchard meadow loss in South Tyrol, Italy**

Alexander Schönafinger^a,b,*^, Lukas Egarter Vigl^a^, Erich Tasser^a^

^a^Institute for Alpine Environment, Eurac Research, Drususallee/Viale Druso 1, I 39100 Bolzano/Bozen, Italy

^b^Department of Ecology, University of Innsbruck, Sternwartestrasse 15/Technikerstraße 25, A 6020 Innsbruck, Austria

*Corresponding author: Alexander Schönafinger, E-mail address: [alexander.schoenafinger@eurac.edu](mailto:alexander.schoenafinger@eurac.edu), Telephone number: +39 0471 055 841

# Supplementary material

## Tables

Table S1 Used agricultural land (UAA: arable land, grassland, permanent crops, without extensively used summer pastures) of South Tyrol and its districts in 1954 and 2010 [1, 2].

|  |  | **UAA (ha)** | | |  |  |
| --- | --- | --- | --- | --- | --- | --- |
|  |  | **1954** |  | **2010** |  | **Change (%)** |
|  |  |  |  |  |  |  |
| Bozen |  | 1,630 |  | 1,747 |  | 7.2 |
| Burggrafenamt |  | 15,172 |  | 15,460 |  | 1.9 |
| Eisacktal |  | 10,878 |  | 9,505 |  | -12.6 |
| Pustertal |  | 23,053 |  | 22,063 |  | -4.3 |
| Salten-Schlern |  | 14,071 |  | 15,269 |  | 8.5 |
| Überetsch-Südtiroler Unterland |  | 13,344 |  | 12,769 |  | -4.3 |
| Vinschgau |  | 12,130 |  | 11,288 |  | -6.9 |
| Wipptal |  | 6,460 |  | 5,661 |  | -12.4 |
|  |  |  |  |  |  |  |
| South Tyrol |  | 96,738 |  | 93,762 |  | -3.1 |

Table S2 Classification of the topographic parameters elevation (a), slope (b), and exposition (c, d).

|  |  | **Class** |  | **Acronym** |  | | **Value range** |
| --- | --- | --- | --- | --- | --- | --- | --- |
|  |  |  |  |  |  | |  |
| **a)** | **Elevation** | [3] | | |  | **(m asl)** | |
|  |  | Colline |  |  |  | | < 800 |
|  |  | Submontane |  |  |  | | 800 - 1.200 |
|  |  | Montane |  |  |  | | 1.200 - 1.800 |
|  |  |  |  |  |  | |  |
| **b)** | **Slope** |  |  |  |  | | **(°)** |
|  |  | Flat |  |  |  | | < 1 |
|  |  | Gentle |  |  |  | | 1 - 11 |
|  |  | Moderate |  |  |  | | 11 - 21 |
|  |  | Steep |  |  |  | | 21 - 31 |
|  |  | Very steep |  |  |  | | > 31 |
|  |  |  |  |  |  | |  |
| **c)** | **Exposition** |  |  |  |  | | **(°)** |
|  |  | North |  |  |  | | 270 - 90 |
|  |  | South |  |  |  | | 90 - 270 |
|  |  |  |  |  |  | |  |
| **d)** | **Exposition** | [4] | | |  | **(°)** | |
|  |  | North |  | N |  | | 337.5 - 22.5 |
|  |  | Northeast |  | NE |  | | 22.5 - 67.5 |
|  |  | East |  | E |  | | 67.5 - 112.5 |
|  |  | Southeast |  | SE |  | | 112.5 - 157.5 |
|  |  | South |  | S |  | | 157.5 - 202.5 |
|  |  | Southwest |  | SW |  | | 202.5 - 247.5 |
|  |  | West |  | W |  | | 247.5 - 292.5 |
|  |  | Northwest |  | NW |  | | 292.5 - 337.5 |

Table S3 Results of the total area of orchard meadows in South Tyrol, categorized by elevation (a), slope (b), and exposition (c, d).

|  |  |  | **Area (ha)** | | |  | **Area (%)** | | |  | **Change (ha)** |  | **Change (%)** |
| --- | --- | --- | --- | --- | --- | --- | --- | --- | --- | --- | --- | --- | --- |
|  |  |  | **1954-56** |  | **2020** |  | **1954-56** |  | **2020** |  |  |  |  |
|  |  |  |  |  |  |  |  |  |  |  |  |  |  |
| a) | Elevation | |  |  |  |  |  |  |  |  |  |  |  |
|  |  | **Colline** | 5,219 |  | 64 |  | 82 |  | 21 |  | -5,155 |  | -98.8 |
|  |  | **Submontane** | 875 |  | 155 |  | 14 |  | 52 |  | -720 |  | -82.3 |
|  |  | **Montane** | 272 |  | 78 |  | 4 |  | 26 |  | -193 |  | -71.2 |
|  |  |  |  |  |  |  |  |  |  |  |  |  |  |
| b) | Slope | |  |  |  |  |  |  |  |  |  |  |  |
|  |  | **Flat** | 459 |  | 7 |  | 7 |  | 2 |  | -453 |  | -98.6 |
|  |  | **Gentle** | 4,387 |  | 74 |  | 69 |  | 25 |  | -4,313 |  | -98.3 |
|  |  | **Moderate** | 1,043 |  | 92 |  | 16 |  | 31 |  | -951 |  | -91.2 |
|  |  | **Steep** | 407 |  | 100 |  | 6 |  | 34 |  | -307 |  | -75.5 |
|  |  | **Very steep** | 68 |  | 24 |  | 1 |  | 8 |  | -44 |  | -64.8 |
|  |  |  |  |  |  |  |  |  |  |  |  |  |  |
| c) | Exposition | |  |  |  |  |  |  |  |  |  |  |  |
|  |  | **South** | 5,035 |  | 216 |  | 79 |  | 73 |  | -4,819 |  | -95.7 |
|  |  | **North** | 1,330 |  | 80 |  | 21 |  | 27 |  | -1,250 |  | -94.0 |
|  |  |  |  |  |  |  |  |  |  |  |  |  |  |
| d) | Exposition | |  |  |  |  |  |  |  |  |  |  |  |
|  |  | **N** | 35 |  | 11 |  | 0.5 |  | 3.7 |  | -23.8 |  | -68.4 |
|  |  | **NE** | 253 |  | 26 |  | 4.0 |  | 8.6 |  | -227.7 |  | -89.9 |
|  |  | **E** | 815 |  | 49 |  | 12.8 |  | 16.6 |  | -765.5 |  | -94.0 |
|  |  | **SE** | 1,897 |  | 52 |  | 29.8 |  | 17.5 |  | -1,844.8 |  | -97.3 |
|  |  | **S** | 1,776 |  | 63 |  | 27.9 |  | 21.4 |  | -1,712.3 |  | -96.4 |
|  |  | **SW** | 643 |  | 57 |  | 10.1 |  | 19.3 |  | -585.6 |  | -91.1 |
|  |  | **W** | 511 |  | 25 |  | 8.0 |  | 8.5 |  | -485.8 |  | -95.1 |
|  |  | **NW** | 440 |  | 13 |  | 6.9 |  | 4.4 |  | -427.4 |  | -97.0 |

Table S4 Orchard meadow area in South Tyrol and its districts in the 1950s and today, their change over time in percent, and the percentage of orchard meadow area (A_OM_), by the used agricultural area (A_UAA_) (Table S1).

|  |  | **Orchard meadow area in:** | | | | |  | **A_OM_ A_UAA_^‑1^ (%) in:** | | |  |
| --- | --- | --- | --- | --- | --- | --- | --- | --- | --- | --- | --- |
|  |  | **1955**  **(ha)** |  | **2020**  **(ha)** |  | **Change**  **(%)** |  | **Historical** |  | **Recent** | |
|  |  |  |  |  |  |  |  |  |  |  | |
| Bozen |  | 320.2 |  | 1.6 |  | - 99.5 |  | 19.6 |  | 0.1 | |
| Burggrafenamt |  | 1,964.1 |  | 65.5 |  | - 96.7 |  | 13.0 |  | 0.4 | |
| Eisacktal |  | 497.4 |  | 41.8 |  | - 91.6 |  | 4.6 |  | 0.4 | |
| Pustertal |  | 250.2 |  | 52.1 |  | - 79.2 |  | 1.1 |  | 0.2 | |
| Salten-Schlern |  | 211.3 |  | 35.6 |  | - 83.1 |  | 1.5 |  | 0.2 | |
| Überetsch - Südtiroler Unterland |  | 1,637.5 |  | 10.2 |  | - 99.4 |  | 12.3 |  | 0.1 | |
| Vinschgau |  | 1,455.8 |  | 84.7 |  | - 94.2 |  | 12.0 |  | 0.8 | |
| Wipptal |  | 28.5 |  | 5.0 |  | - 82.7 |  | 0.4 |  | 0.1 | |
|  |  |  |  |  |  |  |  |  |  |  | |
| South Tyrol |  | 6,364.9 |  | 296.4 |  | - 95.3 |  | 6.6 |  | 0.3 | |

Table S5 Land-use/land-cover (LULC) types in South Tyrol [5] divided into main groups. (*): not relevant for LULC change analysis.

| **LULC groups** | **LULC types** |  |
| --- | --- | --- |
| Built-up area | 11000 - Artificial surfaces and constructions |  |
|  | 11100 - Dense settlement area |  |
|  | 11200 - Low density settlement area |  |
|  | 11300 – Built-up area |  |
|  | 11400 - Open settlement area |  |
|  | 12100 - Industrial and commercial zones |  |
|  | 14100 - Green urban areas |  |
|  | 31450 - Tree cover in urban context |  |
| Infrastructure | 12210 - Roads motorways and trunks |  |
|  | 12220 - Road networks |  |
|  | 12221 - Roads tertiary and others |  |
|  | 12230 - Railways train tracks |  |
|  | 12240 - Unpaved roads and tracks |  |
| Arable land | 21000 - Cultivated areas - Arable land - Annual crops |  |
|  | 21211 - Common wheat |  |
|  | 21213 - Barley |  |
|  | 21214 - Rye |  |
|  | 21215 - Oats | (*) |
|  | 21216 - Maize |  |
|  | 21218 - Triticale | (*) |
|  | 21219 - Other cereals | (*) |
|  | 21221 - Potatoes |  |
|  | 21222 - Sugar beet |  |
|  | 21223 - Other root crops | (*) |
|  | 21230 - Other non-permanent industrial crops | (*) |
|  | 21231 - Sunflower |  |
|  | 21232 - Rape and turnip rape |  |
|  | 21233 - Soya |  |
|  | 21240 - Dry pulses |  |
|  | 21250 - Fodder crops (cereals and leguminous) |  |
|  | 21290 - Bare arable land |  |
| Orchards | 22000 - Permanent crops |  |
|  | 22200 - Orchards |  |
|  | 31400 - Tree cover in agricultural context |  |
| Vineyards | 22100 - Vineyard |  |
| Orchard meadows | 22300 - Orchard Meadow |  |
| Grassland | 23100 - Managed grassland - Pastures |  |
|  | 23200 - Seminatural grassland - Meadows |  |
|  | 32100 - Alpine and sub-alpine natural grassland | (*) |
| Forests | 31100 - Broadleaf tree cover |  |
|  | 31102 - Broadleaf tree cover 30-60% |  |
|  | 31103 - Broadleaf tree cover 60-100% |  |
|  | 31200 - Coniferous tree cover |  |
|  | 31202 - Coniferous tree cover 30-60% |  |
|  | 31203 - Coniferous tree cover 60-100% |  |
|  | 31300 - Mixed tree cover |  |
| Woody features and shrubland | 31500 - Green linear elements - linear woody features |  |
|  | 31600 - Patchy woody features |  |
|  | 31610 - Additional woody features |  |
|  | 32000 - Scrub and shrubland |  |
|  | 32300 - Sclerophyllous vegetation | (*) |
| Wetland | 32200 - Moors and heathland - other scrubland |  |
|  | 41000 - Wetland (permanent wet areas) - inland marshes |  |
| Bare rocks and rock debris | 33100 - Beaches, dunes, sands | (*) |
|  | 33200 - Bare rocks and rock debris |  |
|  | 33300 - Sparsely vegetated land |  |
| Waterbodies | 33500 - Permanent snow covered surfaces | (*) |
|  | 51000 - Water bodies |  |
|  | 51100 – River network |  |
|  | 51200 - Riverbed > 10m width |  |

Table S6 Land use/land cover (LULC) change of orchard meadows in South Tyrol and in its districts since the 1950s. A_OM_: orchard meadow area; A_LUC_: area of LULC change; A_UAA_: used agricultural area in 2010.

|  | **A_OM_**  **(ha)** | **LULC groups** | **A_LUC_**  **(ha)** | **A_LUC_**  **(%)** | **A_UAA_**  **(%)** |
| --- | --- | --- | --- | --- | --- |
| South Tyrol | 6,365 | Orchards | 3,564 | 56.0 | 3.80 |
|  |  | Built-up areas | 908 | 14.3 |  |
|  |  | Infrastructure | 537 | 8.4 |  |
|  |  | Grassland | 520 | 8.2 | 0.55 |
|  |  | Forests | 380 | 6.0 |  |
|  |  | Vineyards | 192 | 3.0 | 0.21 |
|  |  | Arable land | 107 | 1.7 | 0.11 |
|  |  | Woody features and shrubland | 100 | 1.6 |  |
|  |  | Orchard meadows | 33 | 0.5 | 0.04 |
|  |  | Waterbodies | 17 | 0.3 |  |
|  |  | Bare rocks and rock debris | 5 | < 0.1 |  |
|  |  | Wetland | 1 | < 0.1 |  |
| Bozen | 320 | Orchards | 183 | 57.2 | 10.5 |
|  |  | Built-up areas | 89 | 27.8 |  |
|  |  | Infrastructure | 31 | 9.6 |  |
|  |  | Vineyards | 6 | 1.9 | 0.34 |
|  |  | Grassland | 5 | 1.5 | 0.27 |
|  |  | Forests | 3 | 1.0 |  |
|  |  | Arable land | 1 | 0.4 | 0.08 |
|  |  | Waterbodies | < 1 | 0.2 |  |
|  |  | Woody features and shrubland | < 1 | 0.2 |  |
|  |  | Orchard meadows | < 1 | < 0.1 | 0.01 |
|  |  | Wetland | < 1 | < 0.1 |  |
| Burggrafenamt | 1,964 | Orchards | 1,193 | 60.7 | 7.71 |
|  |  | Built-up areas | 287 | 14.6 |  |
|  |  | Grassland | 175 | 8.9 | 1.13 |
|  |  | Infrastructure | 139 | 7.1 |  |
|  |  | Forests | 79 | 4.0 |  |
|  |  | Woody features and shrubland | 28 | 1.4 |  |
|  |  | Vineyards | 25 | 1.3 | 0.16 |
|  |  | Arable land | 22 | 1.1 | 0.15 |
|  |  | Orchard meadows | 9 | 0.5 | 0.06 |
|  |  | Waterbodies | 4 | 0.2 |  |
|  |  | Bare rocks and rock debris | 3 | 0.1 |  |
|  |  | Wetland | < 1 | < 0.1 |  |

continue Table S6

|  | **A_OM_**  **(ha)** | **LULC groups** | **A_LUC_**  **(ha)** | **A_LUC_**  **(%)** | **A_UAA_**  **(%)** |
| --- | --- | --- | --- | --- | --- |
| Eisacktal | 497 | Grassland | 116 | 23.3 | 1.22 |
|  |  | Built-up areas | 105 | 21.1 |  |
|  |  | Orchards | 100 | 20.1 | 1.05 |
|  |  | Forests | 72 | 14.4 |  |
|  |  | Infrastructure | 46 | 9.3 |  |
|  |  | Woody features and shrubland | 24 | 4.9 |  |
|  |  | Arable land | 14 | 2.9 | 0.15 |
|  |  | Vineyards | 13 | 2.6 | 0.14 |
|  |  | Orchard meadows | 4 | 0.8 | 0.04 |
|  |  | Waterbodies | 2 | 0.5 |  |
|  |  | Bare rocks and rock debris | < 1 | 0.1 |  |
|  |  | Wetland | < 1 | < 0.1 |  |
| Pustertal | 250 | Forests | 76 | 30.3 |  |
|  |  | Grassland | 68 | 27.3 | 0.31 |
|  |  | Built-up areas | 30 | 12.2 |  |
|  |  | Arable land | 30 | 12.0 | 0.14 |
|  |  | Woody features and shrubland | 18 | 7.1 |  |
|  |  | Infrastructure | 16 | 6.3 |  |
|  |  | Orchards | 7 | 2.8 | 0.03 |
|  |  | Orchard meadows | 5 | 2.0 | 0.02 |
|  |  | Waterbodies | < 1 | < 0.1 |  |
|  |  | Wetland | < 1 | < 0.1 |  |
|  |  | Bare rocks and rock debris | < 1 | < 0.1 |  |
| Salten-Schlern | 211 | Grassland | 83 | 39.4 | 0.54 |
|  |  | Forests | 60 | 28.2 |  |
|  |  | Built-up areas | 25 | 11.7 |  |
|  |  | Intensive orchards | 16 | 7.5 | 0.10 |
|  |  | Infrastructure | 13 | 6.1 |  |
|  |  | Woody features and shrubland | 6 | 2.8 |  |
|  |  | Vineyards | 3 | 1.6 | 0.02 |
|  |  | Arable land | 3 | 1.5 | 0.02 |
|  |  | Orchard meadows | 2 | 1.1 | 0.02 |
|  |  | Waterbodies | < 1 | < 0.1 |  |
|  |  | Bare rocks and rock debris | < 1 | < 0.1 |  |

continue Table S6

|  | **A_OM_**  **(ha)** | **LULC groups** | **A_LUC_**  **(ha)** | **A_LUC_**  **(%)** | **A_UAA_**  **(%)** |
| --- | --- | --- | --- | --- | --- |
| Überetsch-  Südtiroler Unterland | 1,638 | Intensive orchards | 1,111 | 67.9 | 8.70 |
|  |  | Built-up areas | 161 | 9.8 |  |
|  |  | Vineyards | 138 | 8.4 | 1.08 |
|  |  | Infrastructure | 127 | 7.8 |  |
|  |  | Forests | 43 | 2.6 |  |
|  |  | Grassland | 31 | 1.9 | 0.24 |
|  |  | Arable land | 14 | 0.9 | 0.11 |
|  |  | Woody features and shrubland | 6 | 0.4 |  |
|  |  | Waterbodies | 5 | 0.3 |  |
|  |  | Wetland | < 1 | < 0.1 |  |
|  |  | Bare rocks and rock debris | < 1 | < 0.1 |  |
|  |  | Orchard meadows | < 1 | < 0.1 | < 0.01 |
| Vinschgau | 1,456 | Orchards | 954 | 65.5 | 8.44 |
|  |  | Built-up areas | 208 | 14.3 |  |
|  |  | Infrastructure | 163 | 11.2 |  |
|  |  | Forests | 40 | 2.8 |  |
|  |  | Grassland | 31 | 2.1 | 0.27 |
|  |  | Arable land | 19 | 1.3 | 0.17 |
|  |  | Woody features and shrubland | 16 | 1.1 |  |
|  |  | Orchard meadows | 12 | 0.8 | 0.10 |
|  |  | Vineyards | 7 | 0.5 | 0.06 |
|  |  | Waterbodies | 5 | 0.4 |  |
|  |  | Bare rocks and rock debris | < 1 | < 0.1 |  |
|  |  | Wetland | < 1 | < 0.1 |  |
| Wipptal | 29 | Grassland | 11 | 40.1 | 0.20 |
|  |  | Forests | 7 | 23.8 |  |
|  |  | Built-up areas | 3 | 10.9 |  |
|  |  | Arable land | 2 | 8.4 | 0.04 |
|  |  | Infrastructure | 2 | 5.7 |  |
|  |  | Woody features and shrubland | 2 | 5.3 |  |
|  |  | Orchards | 1 | 5.2 | 0.03 |
|  |  | Orchard meadows | < 1 | 0.6 | < 0.01 |
|  |  | Waterbodies | < 1 | < 0.1 |  |

Table S7 R packages used during the evaluation of spatial and topographic variables within R [6].

| **Statistical packages** | | |  | **Graphical packages** | | |
| --- | --- | --- | --- | --- | --- | --- |
| **Name** |  | **Reference** |  | **Name** |  | **Reference** |
|  |  |  |  |  |  |  |
| tidyverse v1.3.2 |  | [7] |  | tidyverse v1.3.2 |  | [7] |
| psych v2.2.9 |  | [8] |  | ggsignif v0.6.4 |  | [9] |
| car v3.1-1 |  | [10] |  | ggpubr v0.6.0 |  | [11] |
| carData v3.0-5 |  | [12] |  | ggpol v0.0.7 |  | [13] |
|  |  |  |  | ggradar v0.2 |  | [14] |
|  |  |  |  | ggalluvial v0.12.4 |  | [15] |

Table S8 List of several ecosystem services [16] provided in agroforestry systems.

| **Provisioning** | **Regulation & maintenance** | **Cultural** |
| --- | --- | --- |
|  |  |  |
| Fiber [17, 18] | Air quality improvement [18–20] | Aesthetic values [17, 19–23] |
| Fodder [17–20, 22] | Climate regulation [17, 18, 20, 22, 24] | Conservation of many fruit and  crop varieties [20, 25] |
| Food [17–20, 23, 26, 27] | Erosion control [17, 24, 28] | Environmental education [18, 20, 24] |
| Genetic  resources [17, 18, 29] | Flood mitigation [20, 24] | Knowledge of traditional  agricultural practices [17, 21, 29] |
| Timber [18, 19, 22] | Groundwater protection [24, 30] | Recreation & ecotourism [18, 21, 24–28, 31] |
|  | Nutrient cycling [20, 23, 25, 32] | Sense of place [23, 25, 27, 31] |
|  | Pest and disease control [19, 29] | Social relations [24, 25, 33] |
|  | Pollination [22, 23, 31] | Spiritual and religious values [24, 25, 31] |
|  | Provision of habitat &  biodiversity [19, 20, 24–26, 30, 33] |  |
|  | Soil conservation [20, 24, 30] |  |
|  | Storm protection [25] |  |
|  | Water purification [17, 20, 21, 23, 25, 26, 30] |  |

Table S9 Fruit variety list, collected throughout interviews and surveys by Initiative Baumgart (2023) [34] and Schönafinger (2023) [35].

| **Type** | **Species** | **Variety** | **Survey year** |
| --- | --- | --- | --- |
|  |  |  |  |
| poaceous fruit | apple | _indet | 2022 |
| poaceous fruit | apple | Adersleber Kalvill | 2022 |
| poaceous fruit | apple | Alkmene | 2022 |
| poaceous fruit | apple | Ananasrenette | 2022 |
| poaceous fruit | apple | Baumanns Renette | 2022 |
| poaceous fruit | apple | Belamie | 2022 |
| poaceous fruit | apple | Boikenapfel | 2022 |
| poaceous fruit | apple | Boskoop | 2022 |
| poaceous fruit | apple | Bozner Apfel | 2022 |
| poaceous fruit | apple | Braeburn | 2022 |
| poaceous fruit | apple | Brixner Plattling | 2022 |
| poaceous fruit | apple | Canada Renette | 2022 |
| poaceous fruit | apple | Champagner-Renette | 2022 |
| poaceous fruit | apple | Charlamovsky | 2022 |
| poaceous fruit | apple | Cosmic Crisp | 2022 |
| poaceous fruit | apple | Cox Orange | 2022 |
| poaceous fruit | apple | Echter Lederer | 2022 |
| poaceous fruit | apple | Edelböhmer | 2022 |
| poaceous fruit | apple | Elstar | 2022 |
| poaceous fruit | apple | Envy | 2022 |
| poaceous fruit | apple | Florina | 2022 |
| poaceous fruit | apple | Fuji | 2022 |
| poaceous fruit | apple | Gala Permäne | 2022 |
| poaceous fruit | apple | Geheimrat Dr. Oldenburg | 2022 |
| poaceous fruit | apple | Gelber Edelapfel | 2022 |
| poaceous fruit | apple | Golden Canada | 2022 |
| poaceous fruit | apple | Golden Delicious | 2022 |
| poaceous fruit | apple | Granny Smith | 2022 |
| poaceous fruit | apple | Graulinger | 2022 |
| poaceous fruit | apple | Gravensteiner | 2022 |
| poaceous fruit | apple | Guffidauner Süßplattling | 2023 |
| poaceous fruit | apple | Harberts Renette | 2022 |
| poaceous fruit | apple | Jakob Fischer | 2022 |
| poaceous fruit | apple | Jakobiapfel | 2022 |
| poaceous fruit | apple | James Grieve | 2022 |
| poaceous fruit | apple | Jonagold | 2022 |
| poaceous fruit | apple | Kaiser Alexander | 2022 |
| poaceous fruit | apple | Kalterer Böhmer | 2022 |
| poaceous fruit | apple | Köstlicher | 2022 |
| poaceous fruit | apple | Kronprinz Rudolf | 2022 |
| poaceous fruit | apple | Lananer Süßling | 2022 |
| poaceous fruit | apple | Maschanzker | 2022 |
| poaceous fruit | apple | Meraner | 2022 |
| poaceous fruit | apple | Minister von Hammerstein | 2022 |
| poaceous fruit | apple | Ontarioapfel | 2022 |
| poaceous fruit | apple | Pfreisling | 2022 |
| poaceous fruit | apple | Pinova | 2022 |

continue Table S9

| **Type** | **Species** | **Variety** | **Survey year** |
| --- | --- | --- | --- |
|  |  |  |  |
| poaceous fruit | apple | Reanda | 2022 |
| poaceous fruit | apple | Rebella | 2022 |
| poaceous fruit | apple | Red Delicious | 2022 |
| poaceous fruit | apple | Red Topaz | 2022 |
| poaceous fruit | apple | Renette | 2022 |
| poaceous fruit | apple | Rewena | 2022 |
| poaceous fruit | apple | Roter Astrachan | 2022 |
| poaceous fruit | apple | Roter Bellefleur | 2022 |
| poaceous fruit | apple | Roter Boskoop | 2022 |
| poaceous fruit | apple | Roter Eisapfel | 2022 |
| poaceous fruit | apple | Roter Gravensteiner | 2022 |
| poaceous fruit | apple | Roter Jonathan | 2022 |
| poaceous fruit | apple | Roter Mond | 2022 |
| poaceous fruit | apple | Roter Stettiner | 2023 |
| poaceous fruit | apple | Samerling | 2022 |
| poaceous fruit | apple | Scarlett Surprise | 2022 |
| poaceous fruit | apple | Schwarzer Edelapfel | 2022 |
| poaceous fruit | apple | Sommerkönig | 2022 |
| poaceous fruit | apple | Stark Delicious | 2022 |
| poaceous fruit | apple | Steinpepping | 2022 |
| poaceous fruit | apple | Suldener Eisapfel | 2022 |
| poaceous fruit | apple | Summerred | 2022 |
| poaceous fruit | apple | Tiroler Maschanzker | 2022 |
| poaceous fruit | apple | Tiroler Platt-Lederer | 2022 |
| poaceous fruit | apple | Tiroler Spitzlederer | 2022 |
| poaceous fruit | apple | Topaz | 2022 |
| poaceous fruit | apple | Tramin Herbstabpfel | 2022 |
| poaceous fruit | apple | Wagner Renette | 2022 |
| poaceous fruit | apple | Weißapfel | 2022 |
| poaceous fruit | apple | Weißer Astrachan | 2022 |
| poaceous fruit | apple | Weißer Klarapfel | 2022 |
| poaceous fruit | apple | Weißer Rosmarin | 2022 |
| poaceous fruit | apple | Weißer Winter-Calville | 2022 |
| poaceous fruit | apple | Weißer Wintertaffetapfel | 2022 |
| poaceous fruit | apple | Wildling | 2022 |
| poaceous fruit | apple | Winesap | 2022 |
| poaceous fruit | apple | Winterapfel | 2022 |
| poaceous fruit | apple | Wintergoldpermäne | 2022 |
| poaceous fruit | apple | Zierapfel | 2022 |
| poaceous fruit | pear | _indet | 2022 |
| poaceous fruit | pear | Abate | 2022 |
| poaceous fruit | pear | Clapps Liebling | 2022 |
| poaceous fruit | pear | Conference | 2022 |
| poaceous fruit | pear | Dessertnaja | 2022 |
| poaceous fruit | pear | Diels Butterbirne | 2022 |
| poaceous fruit | pear | Echte Rittner Kloatze | 2022 |
| poaceous fruit | pear | Esperens Bergamotte | 2022 |
| poaceous fruit | pear | Ferch-Birne Nr.3 | 2022 |
| poaceous fruit | pear | Frühe von Trevoux | 2022 |
| poaceous fruit | pear | Giffards Butterbirne | 2022 |
| poaceous fruit | pear | Grüne Winterbirne | 2022 |
| poaceous fruit | pear | Gute Luise von Avranches | 2022 |

continue Table S9

| **Type** | **Species** | **Variety** | **Survey year** |
| --- | --- | --- | --- |
|  |  |  |  |
| poaceous fruit | pear | Holzfarbige Butterbirne | 2023 |
| poaceous fruit | pear | Honigbirne | 2022 |
| poaceous fruit | pear | Isebard | 2022 |
| poaceous fruit | pear | Kaiser Alexander | 2022 |
| poaceous fruit | pear | Klotze | 2022 |
| poaceous fruit | pear | Lebruns Butterbirne | 2022 |
| poaceous fruit | pear | Lorenzbirne | 2022 |
| poaceous fruit | pear | Martin Secco | 2022 |
| poaceous fruit | pear | Muskateller Birne | 2022 |
| poaceous fruit | pear | Muterbirn Vellau | 2022 |
| poaceous fruit | pear | Nashi-Birne | 2022 |
| poaceous fruit | pear | Novembra-Nojabrskaja | 2022 |
| poaceous fruit | pear | Oberösterreichische Weinbirne | 2022 |
| poaceous fruit | pear | Palabirne | 2022 |
| poaceous fruit | pear | Pastorenbirne | 2022 |
| poaceous fruit | pear | Pradel | 2022 |
| poaceous fruit | pear | Putzerbirne | 2023 |
| poaceous fruit | pear | Putzerbirne | 2022 |
| poaceous fruit | pear | Rocha | 2022 |
| poaceous fruit | pear | Rote Williams Christ | 2022 |
| poaceous fruit | pear | Schutzengelbirne | 2022 |
| poaceous fruit | pear | Sommerbirne | 2022 |
| poaceous fruit | pear | Stuttgarter Geißhirtle | 2022 |
| poaceous fruit | pear | Teser | 2022 |
| poaceous fruit | pear | Triumph de Vienne | 2022 |
| poaceous fruit | pear | Weidenblättrige Birne | 2022 |
| poaceous fruit | pear | Weisbirne | 2022 |
| poaceous fruit | pear | Williams Christ | 2022 |
| poaceous fruit | pear | Winterbirne | 2022 |
| poaceous fruit | pear | Zitronenbirne | 2022 |
| poaceous fruit | medlar | _indet | 2022 |
| poaceous fruit | medlar | Japanische Wollmispel | 2022 |
| poaceous fruit | medlar | Nespele Tiss | 2022 |
| poaceous fruit | quince | _indet | 2022 |
| poaceous fruit | quince | Apfel-Quitte | 2022 |
| poaceous fruit | quince | Birnen-Quitte | 2022 |
| stone fruit | cherry | _indet | 2022 |
| stone fruit | cherry | Kassins Frühe Herzkirsche | 2022 |
| stone fruit | cherry | Kornelkirsche | 2022 |
| stone fruit | cherry | Regina | 2022 |
| stone fruit | cherry | Sauerkirsche | 2022 |
| stone fruit | cherry | Summit | 2022 |
| stone fruit | cherry | Vogelkirsche | 2022 |
| stone fruit | cherry | Weichselkirsche | 2022 |
| stone fruit | cherry | Zierkirsche | 2022 |
| stone fruit | almond | _indet | 2022 |
| stone fruit | almond | Papierski | 2022 |
| stone fruit | apricot | _indet | 2022 |
| stone fruit | apricot | Aurora | 2022 |
| stone fruit | apricot | Bergeron | 2022 |
| stone fruit | apricot | Clarina | 2022 |
| stone fruit | apricot | Goldrich | 2022 |

continue Table S9

| **Type** | **Species** | **Variety** | **Survey year** |
| --- | --- | --- | --- |
|  |  |  |  |
| stone fruit | apricot | Hargrand | 2022 |
| stone fruit | apricot | Hilde | 2022 |
| stone fruit | apricot | Himalaya-Marille | 2022 |
| stone fruit | apricot | Kuresia | 2022 |
| stone fruit | apricot | Mino | 2022 |
| stone fruit | apricot | Orangered | 2022 |
| stone fruit | apricot | Reale D'Imola | 2022 |
| stone fruit | apricot | Tiroler Spätblüher | 2022 |
| stone fruit | apricot | Tyrinthos | 2022 |
| stone fruit | apricot | Ungarische Beste | 2022 |
| stone fruit | apricot | Vinschger Marille | 2022 |
| stone fruit | peach | _indet | 2022 |
| stone fruit | peach | Benedicte | 2022 |
| stone fruit | peach | Glohaven | 2022 |
| stone fruit | peach | Jayhaven | 2022 |
| stone fruit | peach | Kernechter vom Vorgebirge | 2022 |
| stone fruit | peach | Nektarine | 2022 |
| stone fruit | peach | Royal Summer | 2022 |
| stone fruit | peach | Saturn | 2022 |
| stone fruit | peach | Springcrest | 2022 |
| stone fruit | peach | Suncrest | 2022 |
| stone fruit | plum | _indet | 2022 |
| stone fruit | plum | Anna Späth | 2023 |
| stone fruit | plum | Damaszenerpflaume blau | 2022 |
| stone fruit | plum | Damaszenerpflaume gelb | 2022 |
| stone fruit | plum | Pflaume grün | 2022 |
| stone fruit | plum | Pflaume weiß | 2022 |
| stone fruit | plum | Pfraumen | 2022 |
| stone fruit | plum | Prugna gialla di Don | 2022 |
| stone fruit | plum | Scheißpfleimle/Orschmarterer | 2023 |
| stone fruit | plum | Schöne aus Löwen | 2023 |
| stone fruit | plum | Wildpflaume | 2022 |
| stone fruit | greengage | _indet | 2022 |
| stone fruit | greengage | Ringlo gelb | 2022 |
| stone fruit | greengage | Ringlo rot | 2022 |
| stone fruit | damson | _indet | 2022 |
| stone fruit | damson | Barbianer Zwetschge | 2022 |
| stone fruit | damson | Blue Frost | 2022 |
| stone fruit | damson | Bozner Zwetschge | 2022 |
| stone fruit | damson | Brixner Zwetschge | 2022 |
| stone fruit | damson | Bühler Frühzwetschge | 2022 |
| stone fruit | damson | Fellenberg Zwetschge | 2022 |
| stone fruit | damson | Hauszwetschge | 2022 |
| stone fruit | damson | Hauszwetschge Moar Schmied | 2022 |
| stone fruit | damson | Hauszwetschge Mölten | 2022 |
| stone fruit | damson | Katinka | 2022 |
| stone fruit | damson | Mirabelle | 2022 |
| stone fruit | damson | Mirabelle Bellamira | 2022 |
| stone fruit | damson | Zwetschge Laas | 2022 |
| stone fruit | damson | Zwetschge Nr.3 Eisacktal | 2022 |
| stone fruit | damson | Zwetschge weiß | 2022 |

continue Table S9

| **Type** | **Species** | **Variety** | **Survey year** |
| --- | --- | --- | --- |
|  |  |  |  |
| stone fruit, others | fig | _indet | 2022 |
| stone fruit, others | olive | _indet | 2022 |
| nut fruit | hazelnut | _indet | 2022 |
| nut fruit | chestnut | Edelkastanie | 2022 |
| nut fruit | walnut | _indet | 2022 |
| soft fruit | khaki | Kaki Apfel | 2022 |
| soft fruit | khaki | Kaki Vaniglia | 2022 |
| soft fruit | mulberry | _indet | 2022 |
| soft fruit | pomegranate | _indet | 2022 |

## Figures


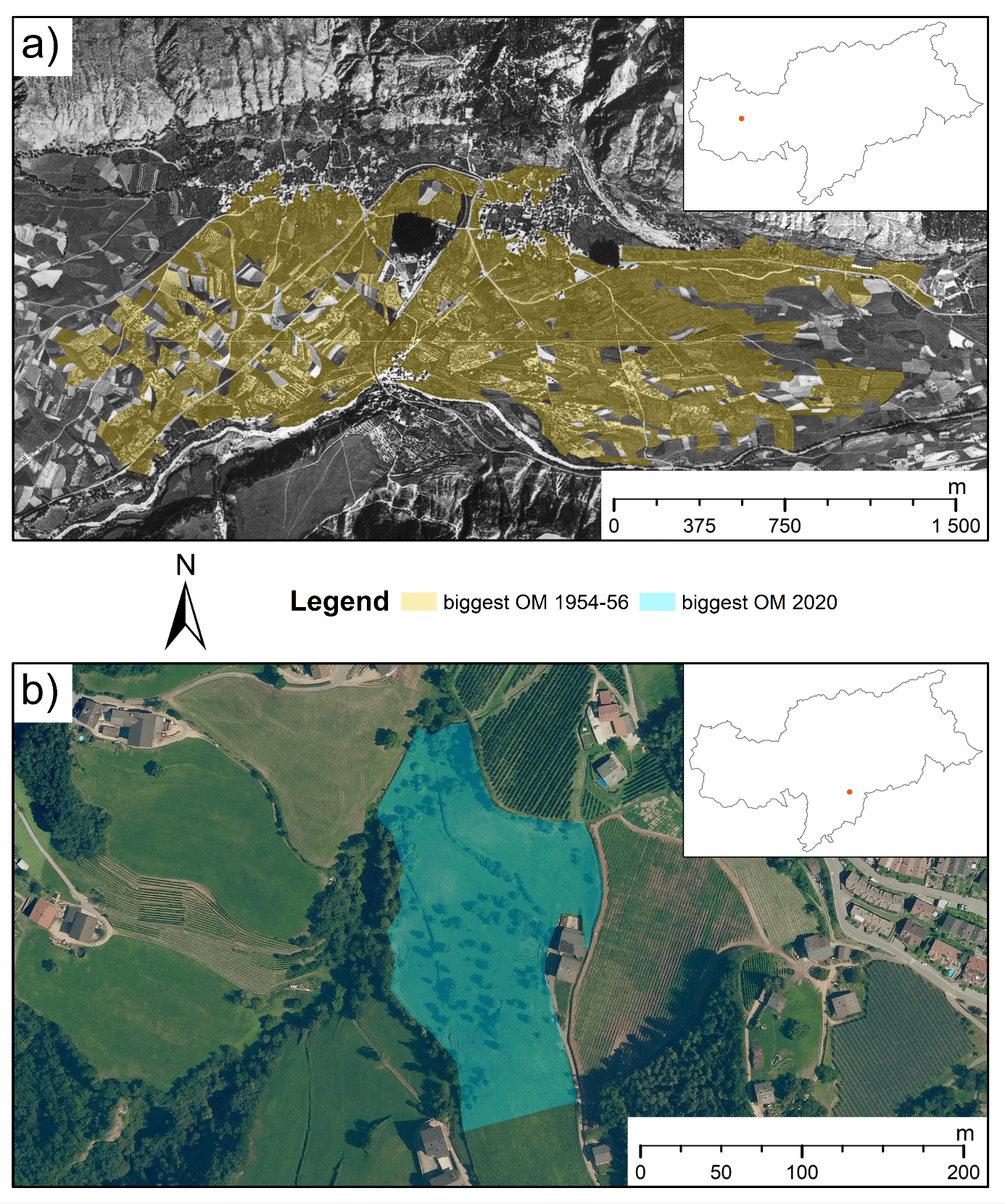


Fig. S1. Biggest contiguous orchard meadow (OM) areas in 1954-56 (Schlanders, Vinschgau; 10.771672°E, 46.627350°N)(a) and 2020 (Völs am Schlern, Salten-Schlern; 11.479478°E, 46.493484°N)(b). The displayed orthophotos are from the years 1954-56 [36] (resolution: 1.5 x 1.5 m)(a) and 2020 [36] (resolution: 0.2 x 0.2 m)(b). The compilation of maps was generated using ArcGis Pro 3.3.1 [37].


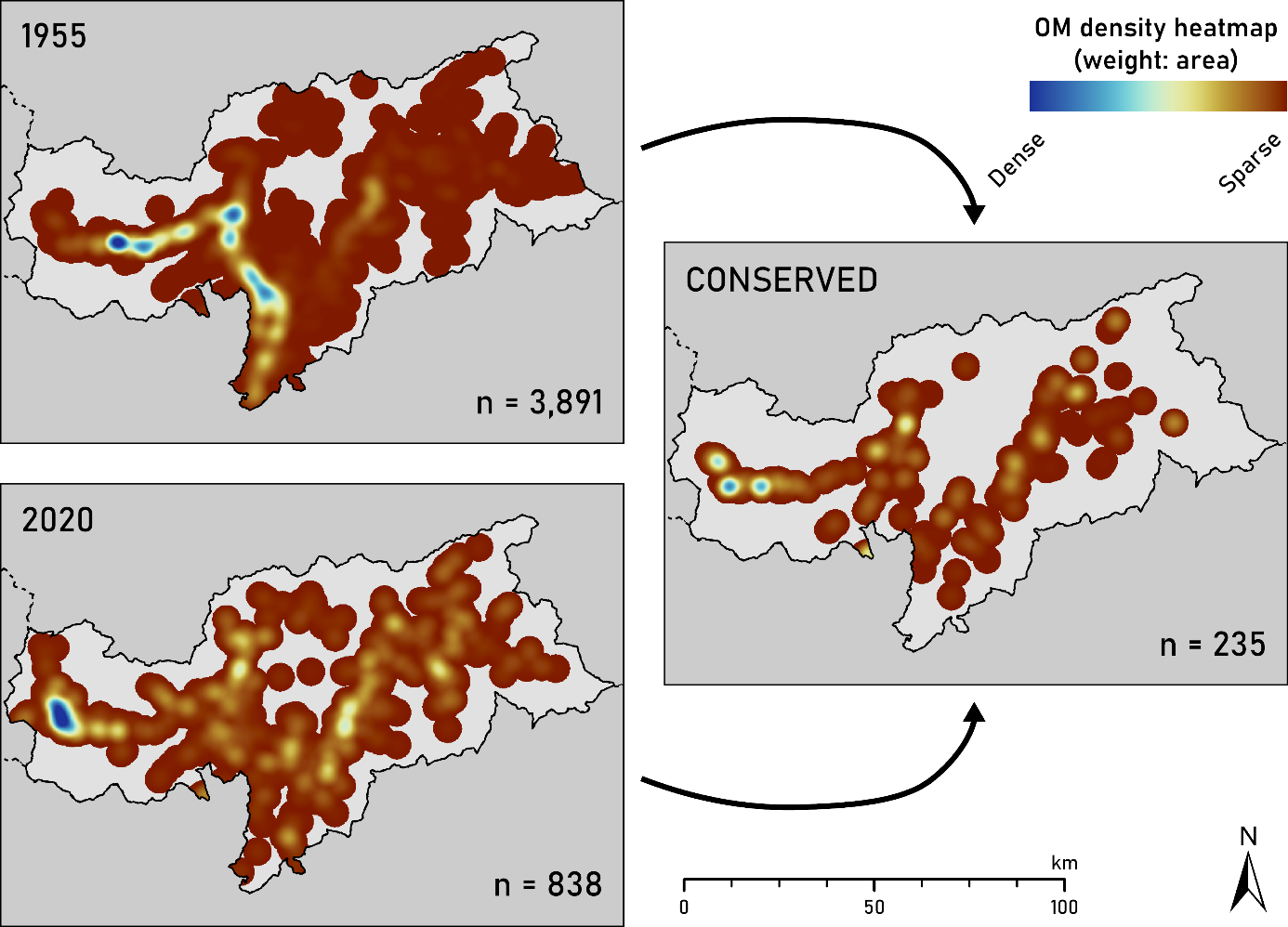


Fig. S2. Relative point density heatmap of orchard meadows (OM) in 1955, 2020 and of conserved orchard meadows in South Tyrol, weighted by area. The conserved map segment shows the orchard meadows present in both 1955 and 2020. The orchard meadow count (n) is displayed at the bottom right corner of each map segment respectively (ArcGis Pro 3.3.1 [37] sympology setting: Type = Heatmap; method = constant; radius = 10 screen unit points; weight field = area; color scheme = roma; rendering quality = best).


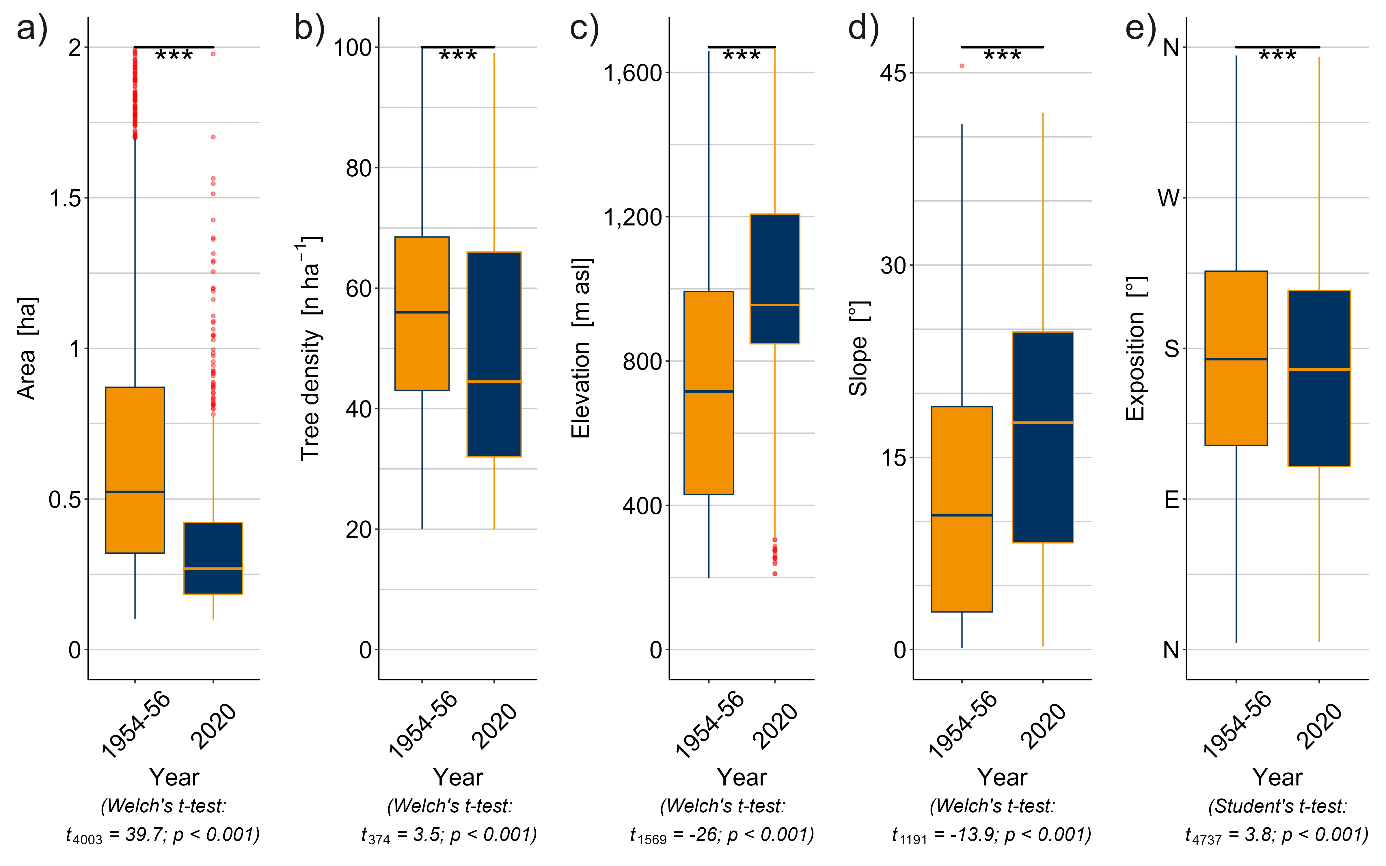


Fig. S3. Orchard meadow distribution in South Tyrol by average area (a), tree density (b), elevation (c), slope (d), and exposition (e).


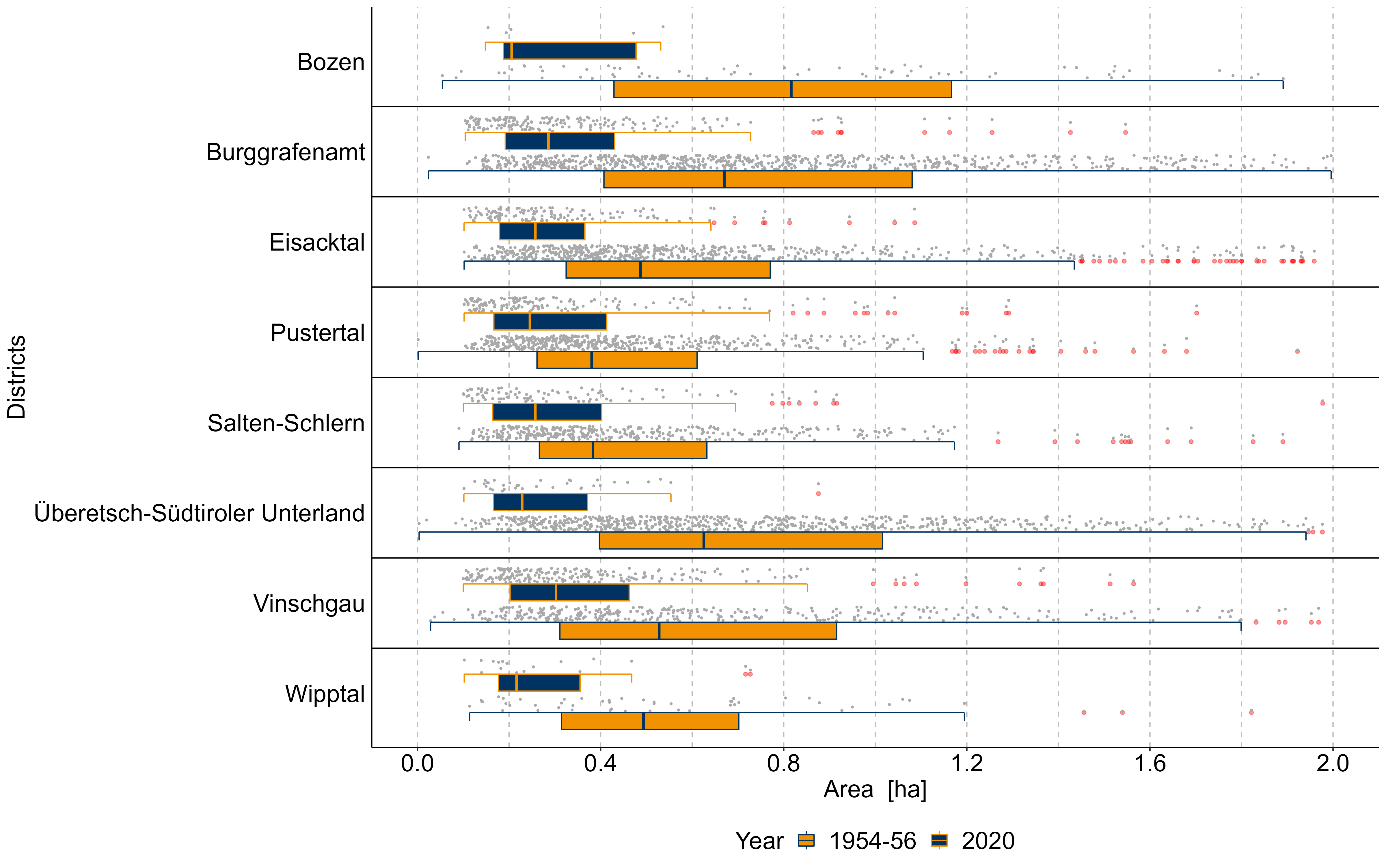


Fig. S4. Orchard meadows in South Tyrol’s districts by average area and year.


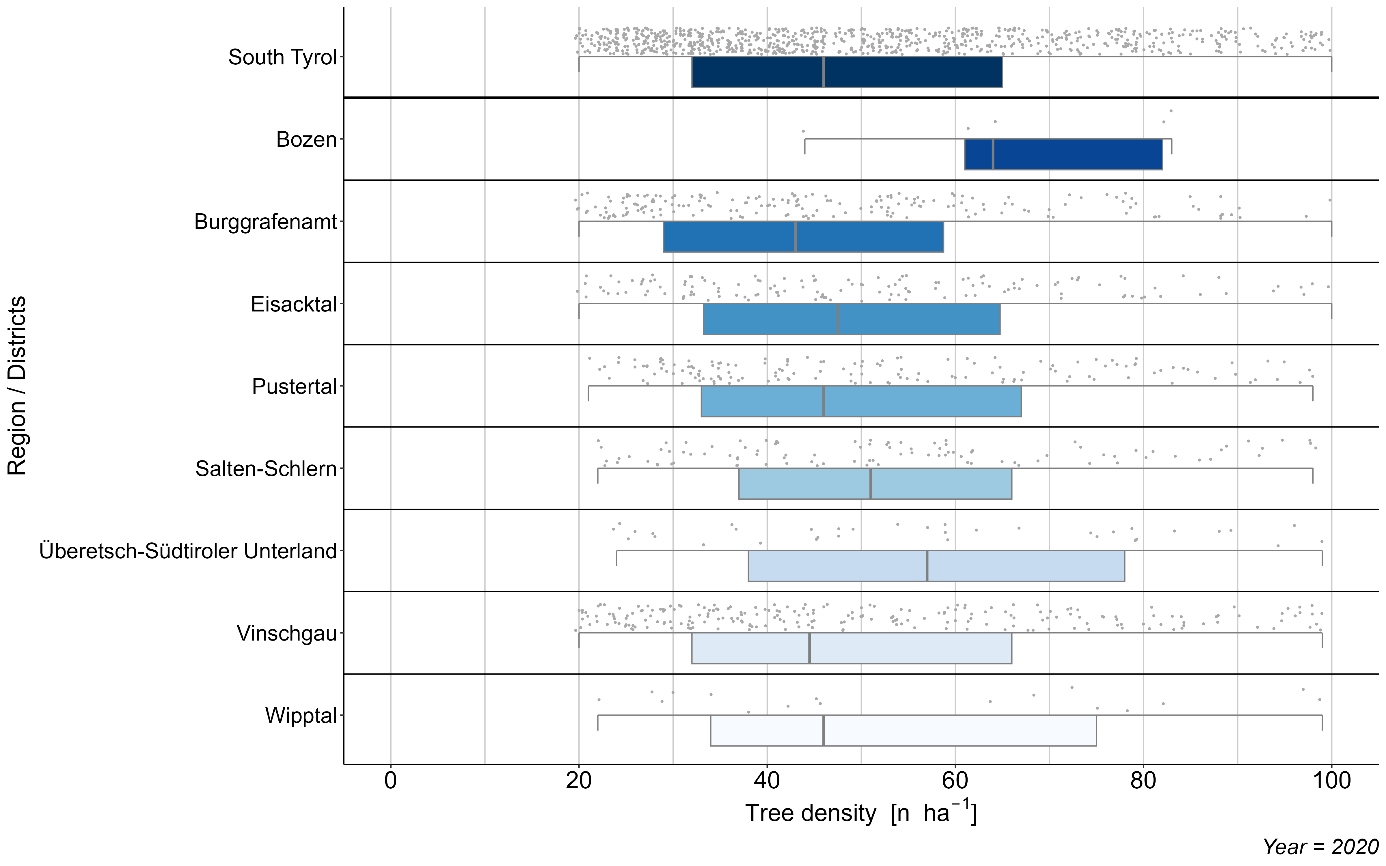


Fig. S5. Orchard meadows in South Tyrol and its districts by average tree density.


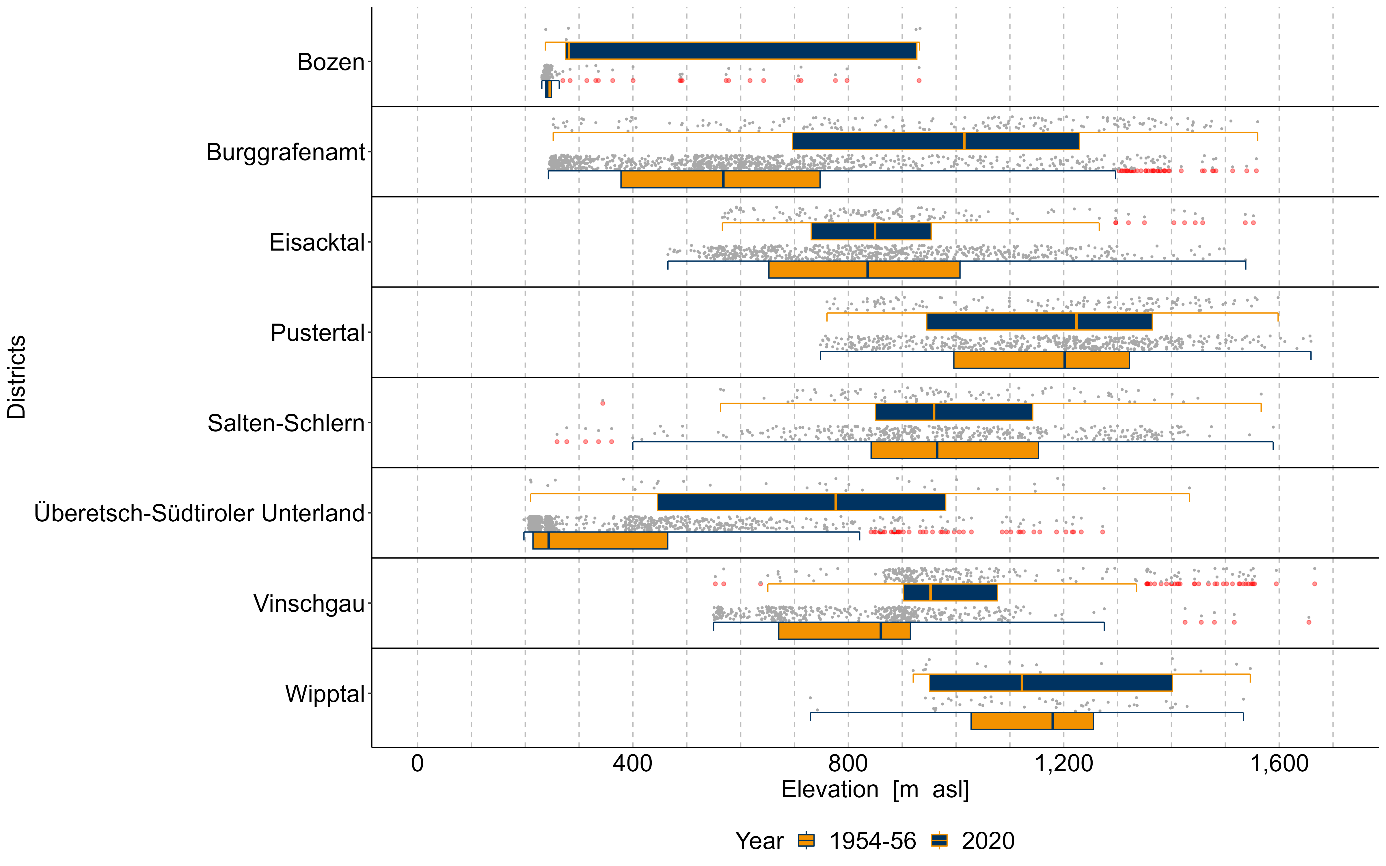


Fig. S6. Orchard meadows in South Tyrol’s districts by average elevation and year.


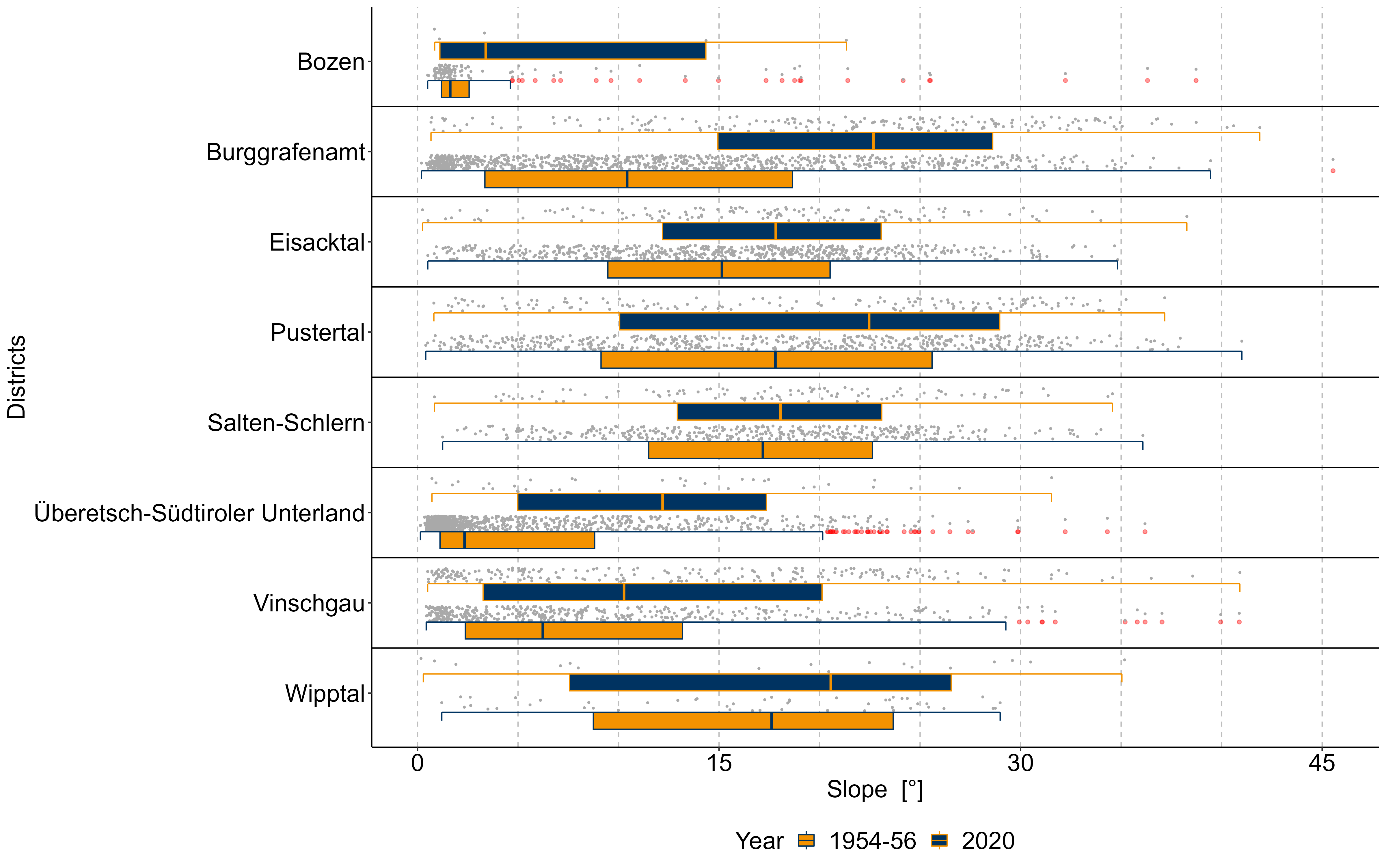


Fig. S7. Orchard meadows in South Tyrol’s districts by average slope and year.


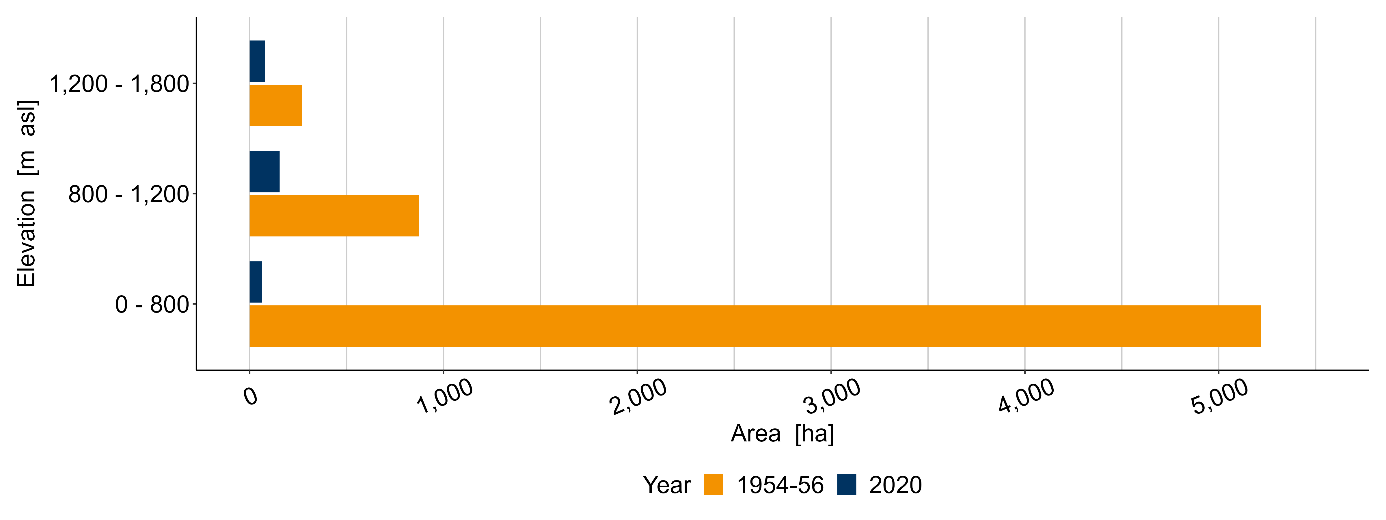


Fig. S8. Total area of orchard meadows in South Tyrol categorized by elevation classes (Table S2a).


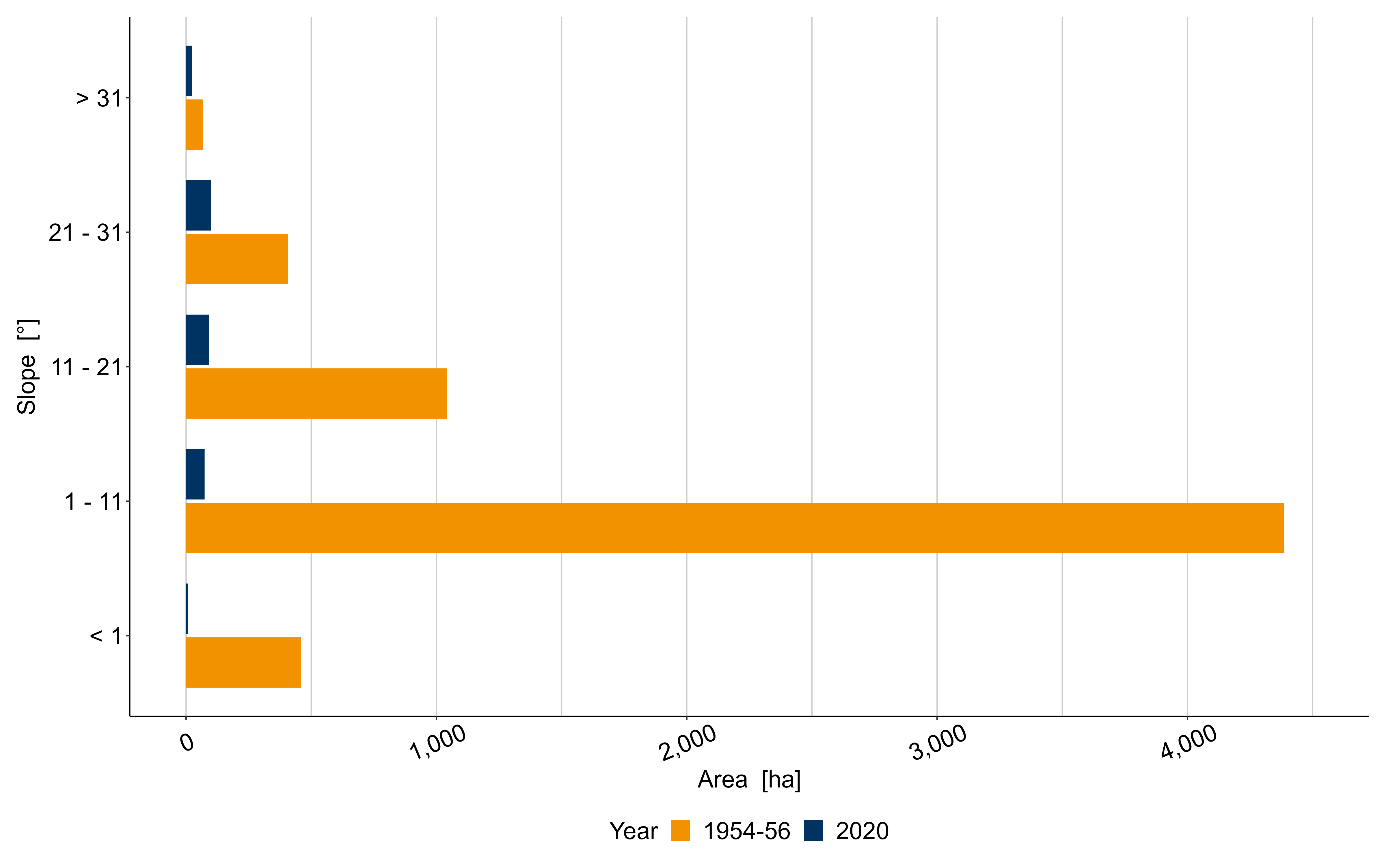


Fig. S9. Total area of orchard meadows in South Tyrol categorized by slope classes (Table S2b).


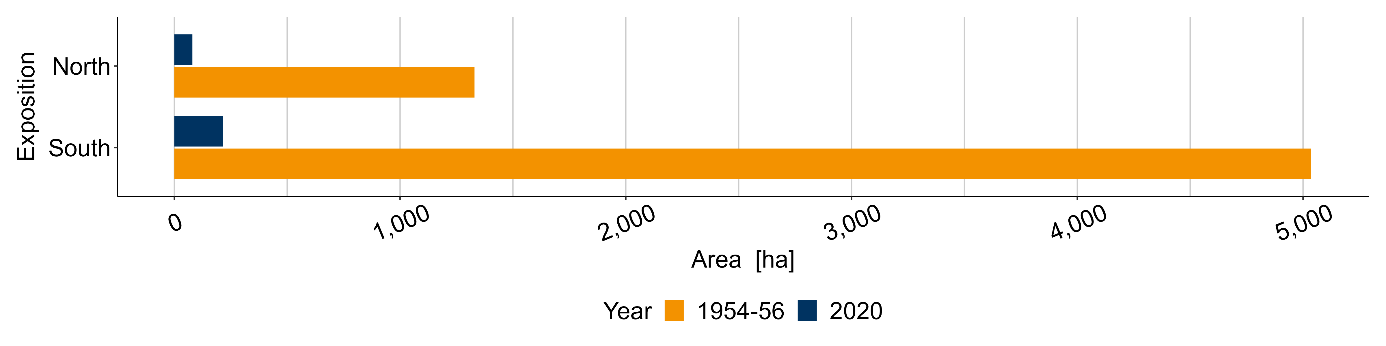


Fig. S10. Total area of orchard meadows in South Tyrol categorized by exposition towards north or south (Table S2c).


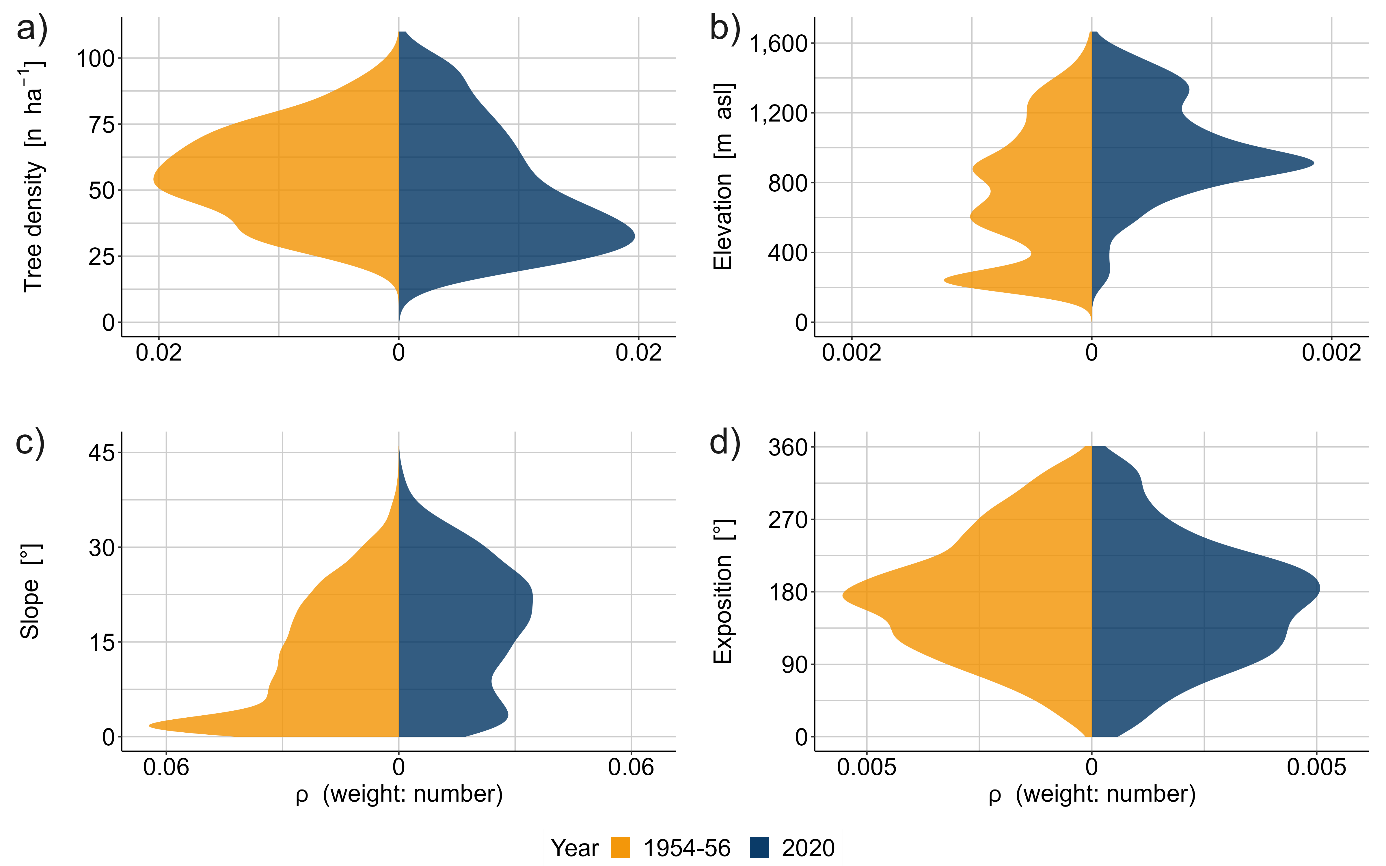


Fig. S11. Temporal Comparison of tree density (only for the district of Vinschgau)(a), elevation (b), slope (c), and aspect (d) of orchard meadows in South Tyrol (Italy), weighted by number.


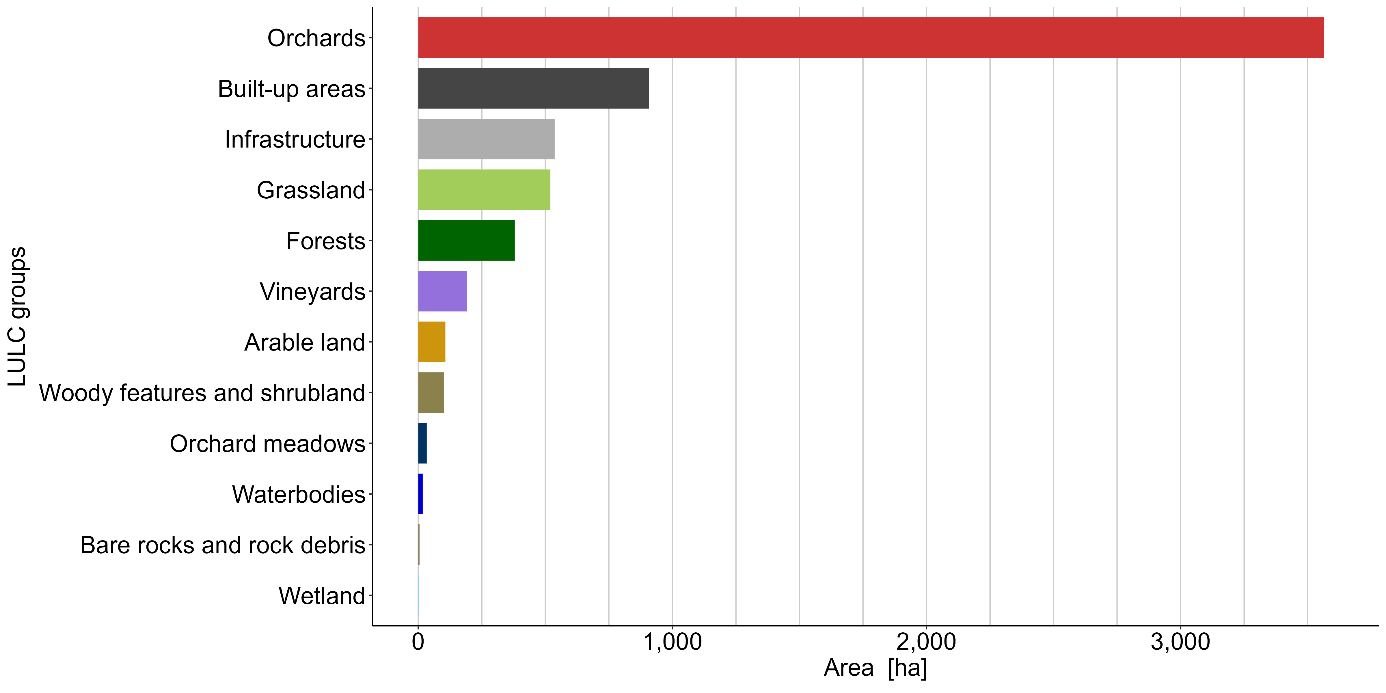


Fig. S12. Current land use/land cover (LULC) on the orchard meadows in the 1950s in South Tyrol.

# References

1. ASTAT (2023) Land- und Forstwirtschaft. https://astat.provinz.bz.it/de/land-forstwirtschaft.asp. Accessed 6 Feb 2024

2. ISTAT (2023) Permanent Census. In: Ist. Naz. Stat. https://www.istat.it/en/censuses. Accessed 24 Feb 2023

3. Autonome Provinz Bozen, Abteilung Forstwirtschaft, Amt für Forstplanung (eds) (2010) Waldtypen, Wuchsgebiete, Bestimmungsschlüssel. Autonome Provinz Bozen

4. Burrough PA, McDonnell R, Lloyd CD (2015) Principles of geographical information systems, Third edition. Oxford University Press, Oxford ; New York

5. Marsoner T, Simion H, Giombini V, Egarter Vigl L, Candiago S (2023) A detailed land use/land cover map for the European Alps macro region. Sci Data 10:468

6. R Core Team (2023) R v4.3.1: A language and Environment for Statistical Computing.

7. Wickham H, Averick M, Bryan J, et al (2019) Welcome to the Tidyverse. J Open Source Softw 4:1686

8. Revelle W (2024) psych v2.4.1: Procedures for Psychological, Psychometric, and Personality Research.

9. Ahlmann-Eltze C, Patil I (2021) ggsignif v0.6.4: R Package for Displaying Significance Brackets for “ggplot2.” https://doi.org/10.31234/osf.io/7awm6

10. Fox J, Weisberg S (2019) An R companion to applied regression, Third edition. SAGE, Los Angeles London New Delhi Singapore Washington, DC Melbourne

11. Kassambara A (2023) ggpubr v0.6.0: “ggplot2” Based Publication Ready Plots.

12. Fox J, Weisberg S, Price B (2022) carData v3.0-5: Companion to Applied Regression Data Sets.

13. Tiedemann H (2022) ggpol v0.0.7: Visualizing Social Science Data with “ggplot2.”

14. Bion R (2022) ggradar v0.2.

15. Brunson C (2020) corybrunson/ggalluvial v0.12.5: remove plyr dependency. https://doi.org/10.5281/ZENODO.3836748

16. Haines-Young R, Potschin M (2018) Common International Classification of Ecosystem Services (CICES) V5.1: Guidance on the Application of the Revised Structure. 53

17. Plieninger T, Levers C, Mantel M, Costa A, Schaich H, Kuemmerle T (2015) Patterns and Drivers of Scattered Tree Loss in Agricultural Landscapes: Orchard Meadows in Germany (1968-2009). PLOS ONE 10:e0126178

18. Rolo V, Hartel T, Aviron S, et al (2020) Challenges and innovations for improving the sustainability of European agroforestry systems of high nature and cultural value: stakeholder perspectives. Sustain Sci 15:1301–1315

19. López-Sánchez A, Perea R, Roig S, Isselstein J, Schmitz A (2020) Challenges on the conservation of traditional orchards: Tree damage as an indicator of sustainable grazing. J Environ Manage 257:110010

20. Nair PKR, Gordon AM, Rosa Mosquera-Losada M (2008) Agroforestry. In: Jørgensen SE, Fath BD (eds) Encycl. Ecol. Elsevier, pp 101–110

21. Fontana V, Radtke A, Bossi Fedrigotti V, Tappeiner U, Tasser E, Zerbe S, Buchholz T (2013) Comparing land-use alternatives: Using the ecosystem services concept to define a multi-criteria decision analysis. Ecol Econ 93:128–136

22. Kornprobst M (1994) Landschaftspflegekonzept Bayern. 2,5: Lebensraumtyp Streuobst. Bayerisches Staatsministerium für Landesentwicklung und Umweltfragen, München in Zsarb. mit der Bayerischen Akademie für Naturschutz und Landschaftspflege (ANL), München

23. Zerbe S (2019) Renaturierung von Ökosystemen im Spannungsfeld von Mensch und Umwelt: ein interdisziplinäres Fachbuch. https://doi.org/10.1007/978-3-662-58650-1

24. Plieninger T, Bieling C, Ohnesorge B, Schaich H, Schleyer C, Wolff F (2013) Exploring Futures of Ecosystem Services in Cultural Landscapes through Participatory Scenario Development in the Swabian Alb, Germany. Ecol Soc 18:art39

25. Fagerholm N, Torralba M, Burgess PJ, Plieninger T (2016) A systematic map of ecosystem services assessments around European agroforestry. Ecol Indic 62:47–65

26. Forejt M, Syrbe R-U (2019) The current status of orchard meadows in Central Europe: Multi-source area estimation in Saxony (Germany) and the Czech Republic. Morav Geogr Rep 27:217–228

27. Schönhart M, Schauppenlehner T, Schmid E, Muhar A (2011) Analysing the maintenance and establishment of orchard meadows at farm and landscape levels applying a spatially explicit integrated modelling approach. J Environ Plan Manag 54:115–143

28. Guariento E, Colla F, Steinwandter M, Plunger J, Tappeiner U, Seeber J (2020) Management Intensification of Hay Meadows and Fruit Orchards Alters Soil Macro- Invertebrate Communities Differently. Agronomy 10:767

29. Plieninger T (2012) Monitoring directions and rates of change in trees outside forests through multitemporal analysis of map sequences. Appl Geogr 32:566–576

30. Nerlich K, Graeff-Hönninger S, Claupein W (2013) Agroforestry in Europe: a review of the disappearance of traditional systems and development of modern agroforestry practices, with emphasis on experiences in Germany. Agrofor Syst 87:475–492

31. Herzog F (1998) *Streuobst*: a traditional agroforestry system as a model for agroforestry development in temperate Europe. Agrofor Syst 42:61–80

32. Plieninger T, Hartel T, Martín-López B, Beaufoy G, Bergmeier E, Kirby K, Montero MJ, Moreno G, Oteros-Rozas E, Van Uytvanck J (2015) Wood-pastures of Europe: Geographic coverage, social–ecological values, conservation management, and policy implications. Biol Conserv 190:70–79

33. Guariento E, Obwegs L, Anderle M, et al (2024) Meadow orchards as a good practice example for improving biodiversity in intensive apple orchards. Biol Conserv 299:110815

34. Initiative Baumgart (2023) Streuobstwiesen in Südtirol. In: Vielfalt Erhalt. https://www.baumgart.it/. Accessed 7 Feb 2024

35. Schönafinger A (2023) Orchard Meadows in South Tyrol: Spatio-temporal development and agro-ecological evaluation. master thesis, University of Innsbruck

36. Autonome Provinz Bozen (2024) GeoKatalog. In: Südtiroler Bürgernetz. http://geokatalog.buergernetz.bz.it/geokatalog/#! Accessed 19 Feb 2024

37. ESRI (2023) ArcGIS Pro.
